# Author Correction: Domain-substituted IGF2 tag modulates targeting of lentiviral gene therapy for Hunter syndrome

**DOI:** 10.1038/s44321-026-00492-8

**Published:** 2026-07-29

**Authors:** Fabio Catalano, Dejan Stevic, Giacomo Zundo, Tessa F Huizer, Zina Dammou, Eva C Vlaar, Drosos Katsavelis, Jeroen C van den Bosch, Hannerieke J M P van den Hout, Esmeralda Oussoren, Ans T van der Ploeg, George J G Ruijter, Gerben Schaaf, W W M Pim Pijnappel

**Affiliations:** 1 Department of Clinical Genetics, Erasmus MC University Medical Center, Rotterdam, 3015GE The Netherlands; 2 Department of Pediatrics, Erasmus MC University Medical Center, Rotterdam, 3015GE The Netherlands; 3 Center for Lysosomal and Metabolic Diseases, Erasmus MC University Medical Center, Rotterdam, 3015GE The Netherlands

## Abstract

**Figure:**
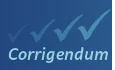

**Correction to:** *EMBO Molecular Medicine* (2025) 17:3197–3226. https://doi.org/10.1038/s44321-025-00314-3 | Published online 29 September 2025

**The corresponding author information is updated**.

The corresponding author information is corrected from:

Correspondence should be addressed to Dr. W.W.M. Pim Pijnappel (w.pijnappel@erasmusmc.nl), Erasmus MC University Medical Center, 3015GE Rotterdam, The Netherlands.

To: (Additions in bold.)

Correspondence should be addressed to Dr. Fabio Catalano (f.catalano@erasmusmc.nl) and Dr. W.W.M. Pim Pijnappel (w.pijnappel@erasmusmc.nl), Erasmus MC University Medical Center, 3015GE Rotterdam, The Netherlands.

All authors agree to this author correction.

