# Supplementary figures and images for: Domain-substituted IGF2 tag modulates targeting of lentiviral gene therapy for Hunter syndrome (part 1 of 2)
Source: EMBO Mol Med. 2025 Sep 29;17(11):3197–226. doi: 10.1038/s44321-025-00314-3 (PMC12603107; doi:10.1038/s44321-025-00314-3)

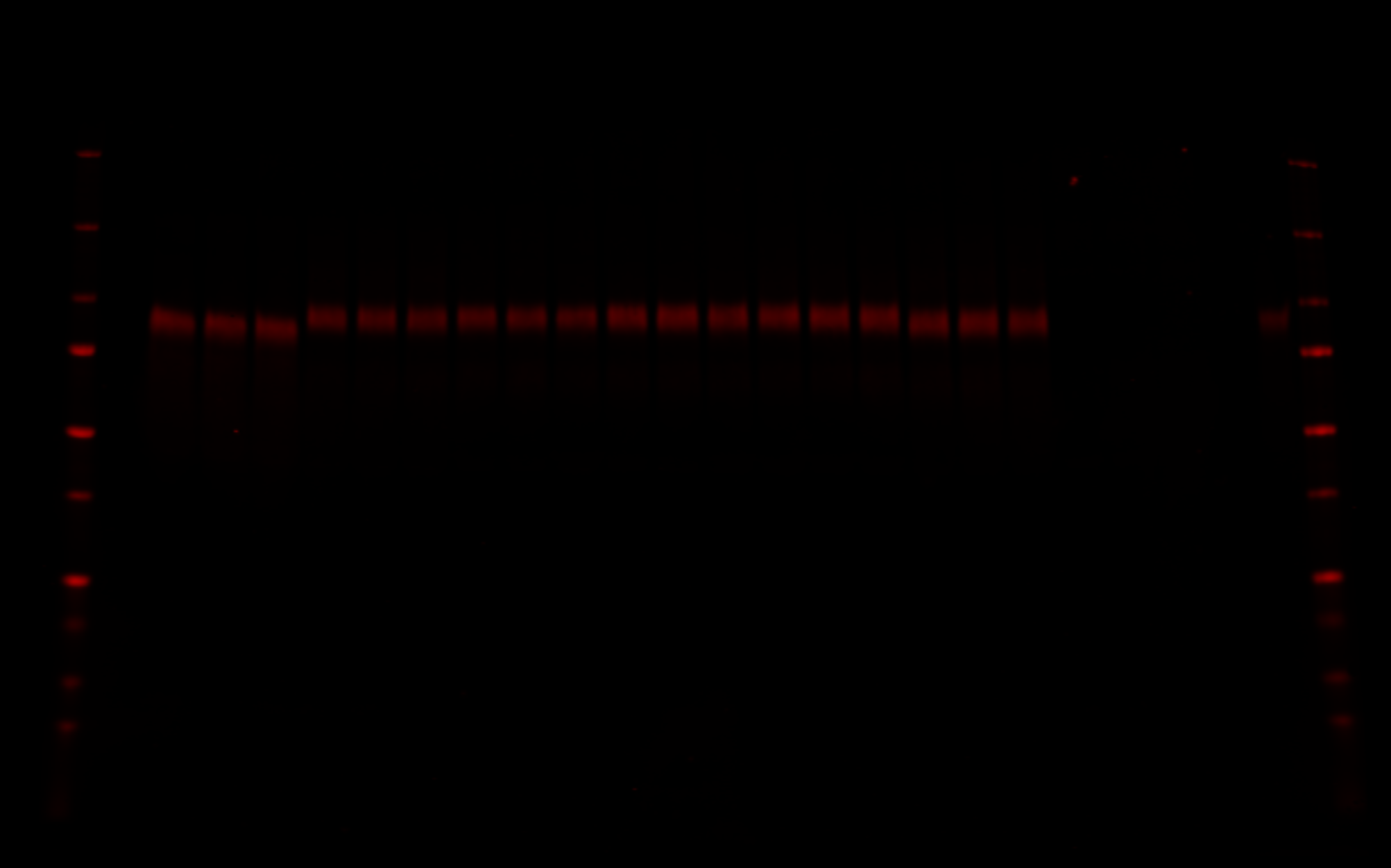

Supplement: Supplementary file 4 — Source data Fig. 1 [file 44321_2025_314_MOESM4_ESM.zip › Figure 1/1C/Source image 1C_Westernblot Transduction HMC3_medium.tif]

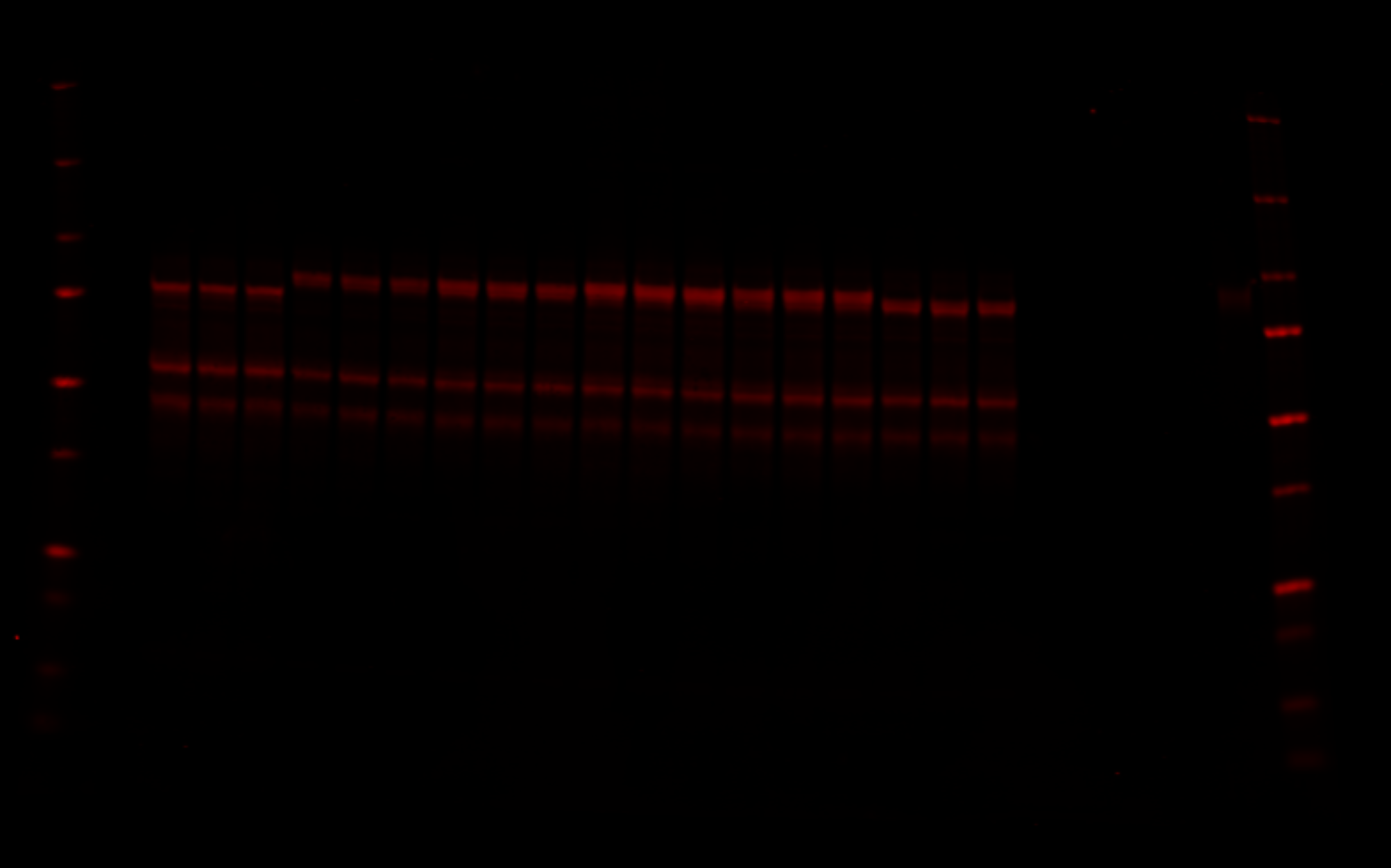

Supplement: Supplementary file 4 — Source data Fig. 1 [file 44321_2025_314_MOESM4_ESM.zip › Figure 1/1C/Source image 1C_Westernblot Transduction HMC3_cells.tif]

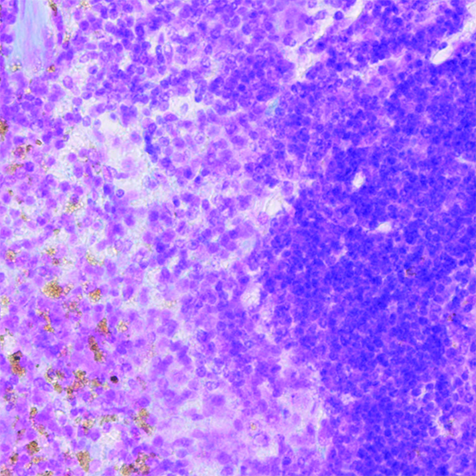

Supplement: Supplementary file 7 — Source data Fig. 4 [file 44321_2025_314_MOESM7_ESM.zip › Figure 4/4A/B8-WT_Spleen.tif]

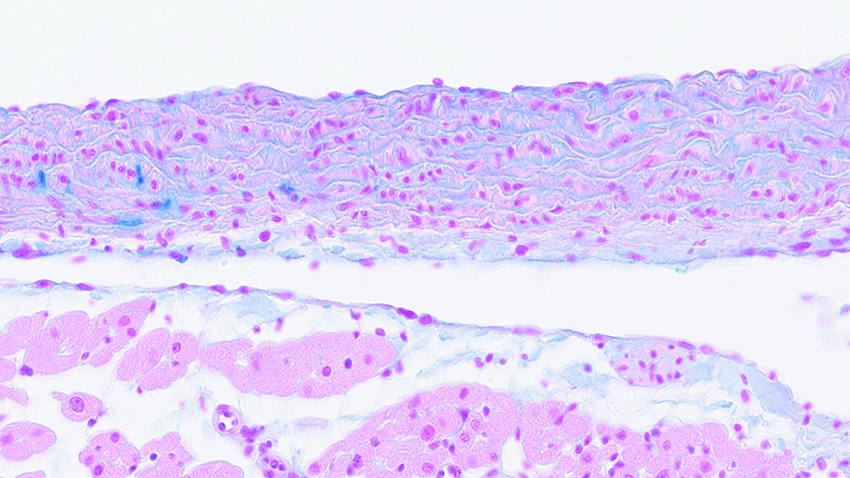

Supplement: Supplementary file 7 — Source data Fig. 4 [file 44321_2025_314_MOESM7_ESM.zip › Figure 4/4A/D4_IDS.SWAP-ApoEco.tif]

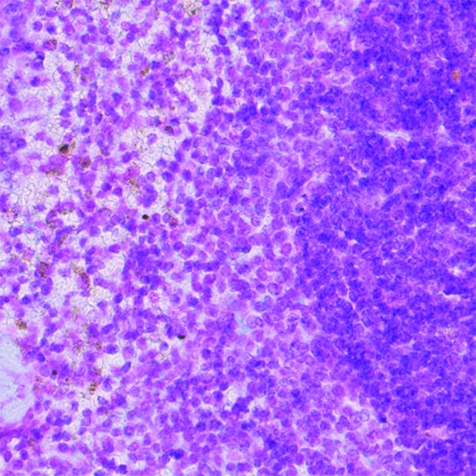

Supplement: Supplementary file 7 — Source data Fig. 4 [file 44321_2025_314_MOESM7_ESM.zip › Figure 4/4A/B2-IDS.IGF2co_Spleen.tif]

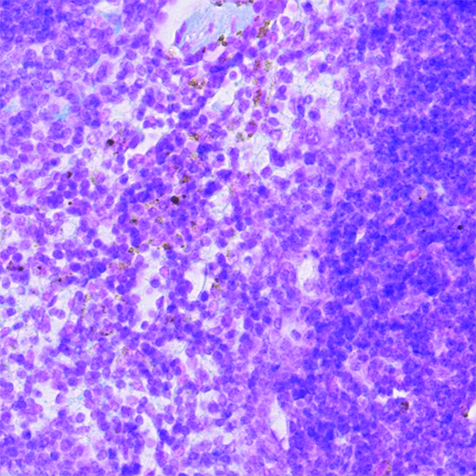

Supplement: Supplementary file 7 — Source data Fig. 4 [file 44321_2025_314_MOESM7_ESM.zip › Figure 4/4A/B5-IDS.SWAP-RAP12x2co_Spleen.tif]

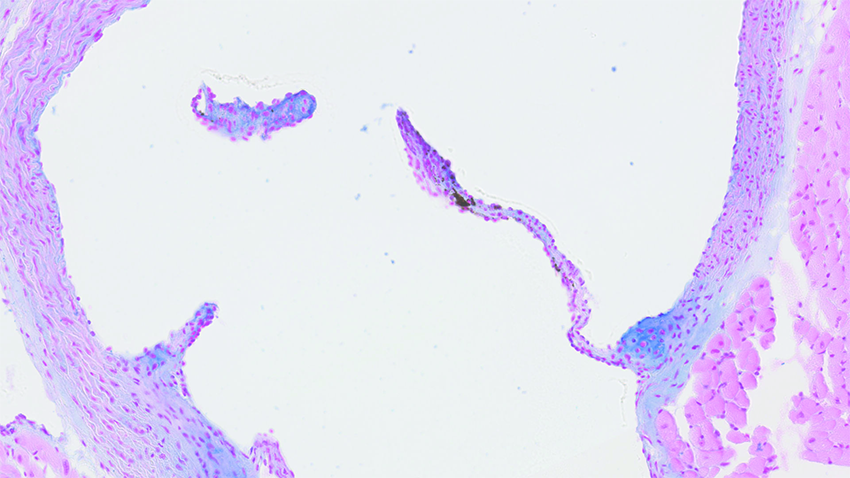

Supplement: Supplementary file 7 — Source data Fig. 4 [file 44321_2025_314_MOESM7_ESM.zip › Figure 4/4A/C7-WT_AV.tif]

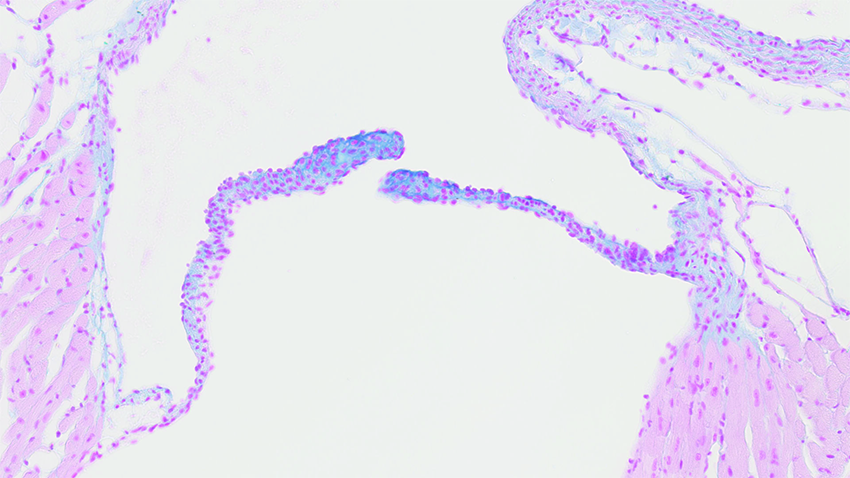

Supplement: Supplementary file 7 — Source data Fig. 4 [file 44321_2025_314_MOESM7_ESM.zip › Figure 4/4A/C5-IDS.SWAP-RAP12x2co_AV.tif]

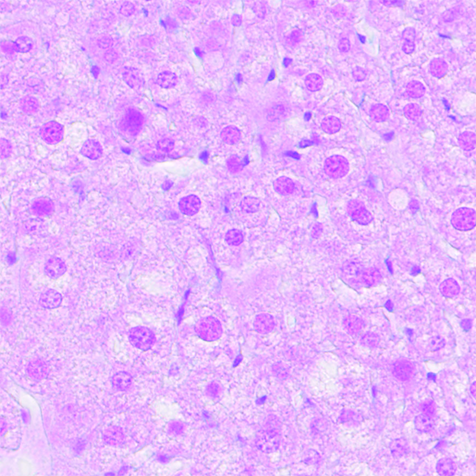

Supplement: Supplementary file 7 — Source data Fig. 4 [file 44321_2025_314_MOESM7_ESM.zip › Figure 4/4A/A8-WT.tif]

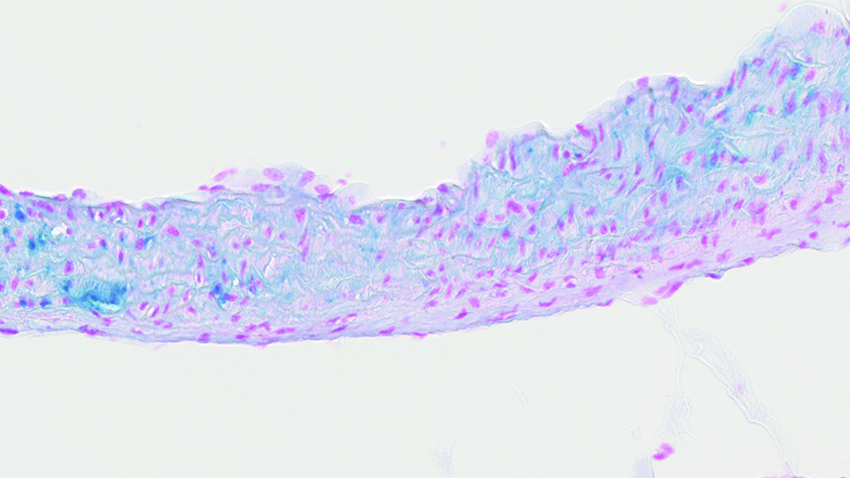

Supplement: Supplementary file 7 — Source data Fig. 4 [file 44321_2025_314_MOESM7_ESM.zip › Figure 4/4A/D5_IDS.SWAP-RAP12x2co.tif]

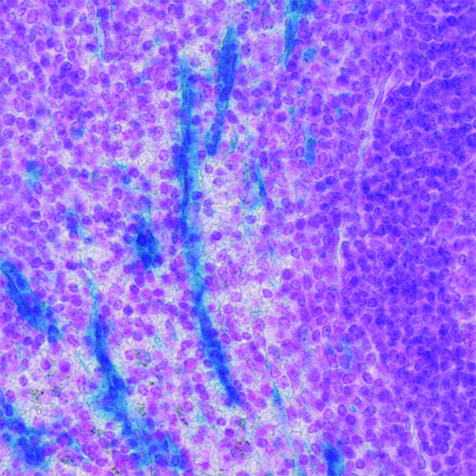

Supplement: Supplementary file 7 — Source data Fig. 4 [file 44321_2025_314_MOESM7_ESM.zip › Figure 4/4A/B7-MPS II_Spleen.tif]

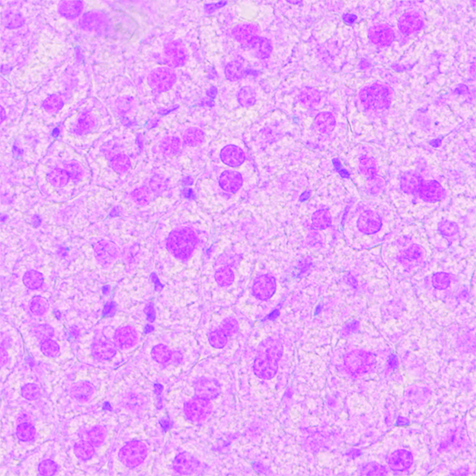

Supplement: Supplementary file 7 — Source data Fig. 4 [file 44321_2025_314_MOESM7_ESM.zip › Figure 4/4A/A3-IDS.IGF2del_co_Liver.tif]

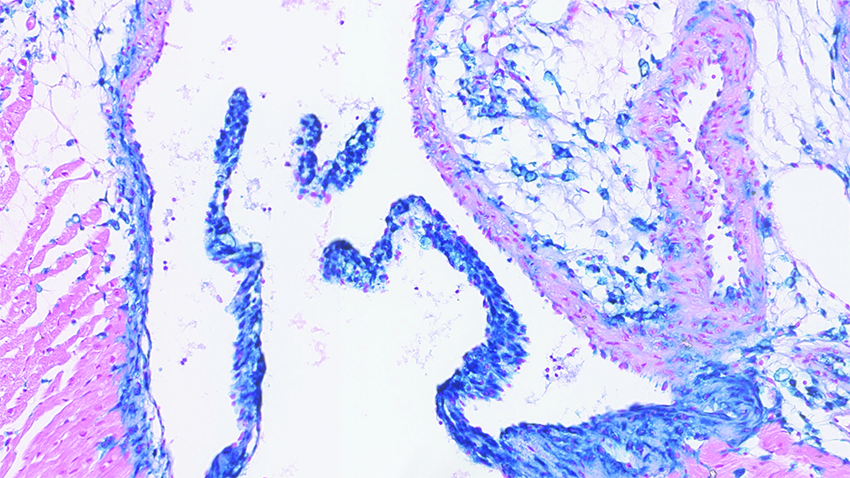

Supplement: Supplementary file 7 — Source data Fig. 4 [file 44321_2025_314_MOESM7_ESM.zip › Figure 4/4A/C6-GFP_AV.tif]

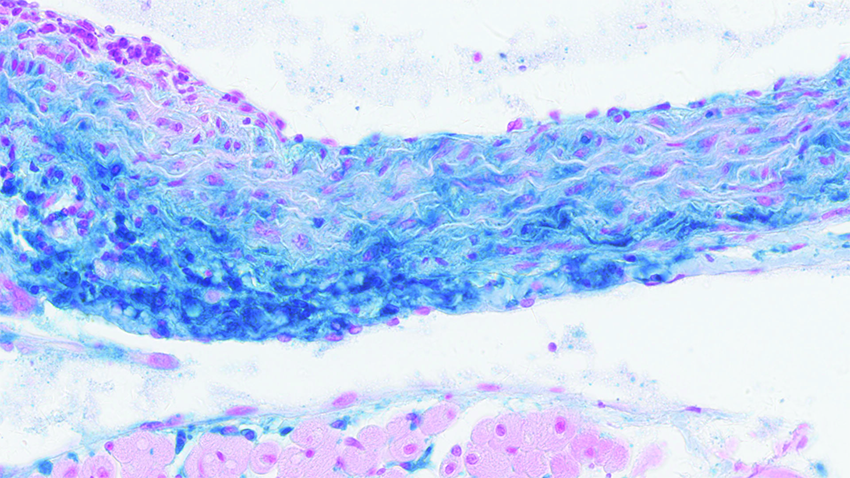

Supplement: Supplementary file 7 — Source data Fig. 4 [file 44321_2025_314_MOESM7_ESM.zip › Figure 4/4A/D6_GFP.tif]

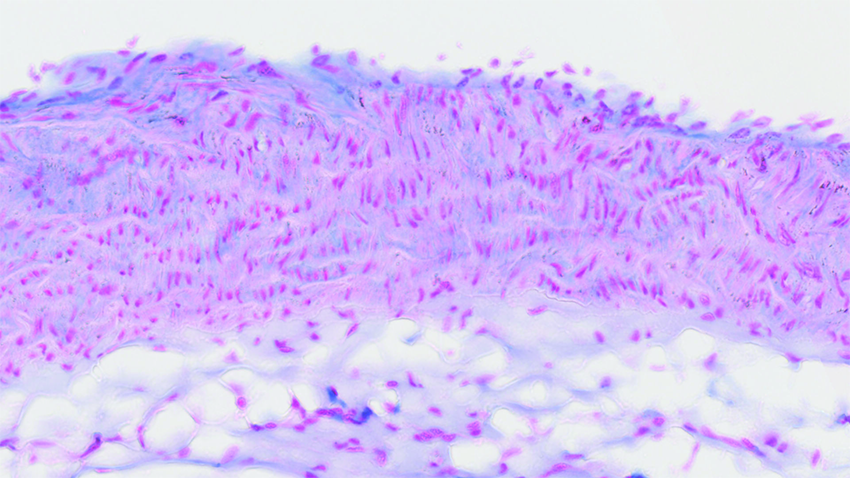

Supplement: Supplementary file 7 — Source data Fig. 4 [file 44321_2025_314_MOESM7_ESM.zip › Figure 4/4A/D8_WT.tif]

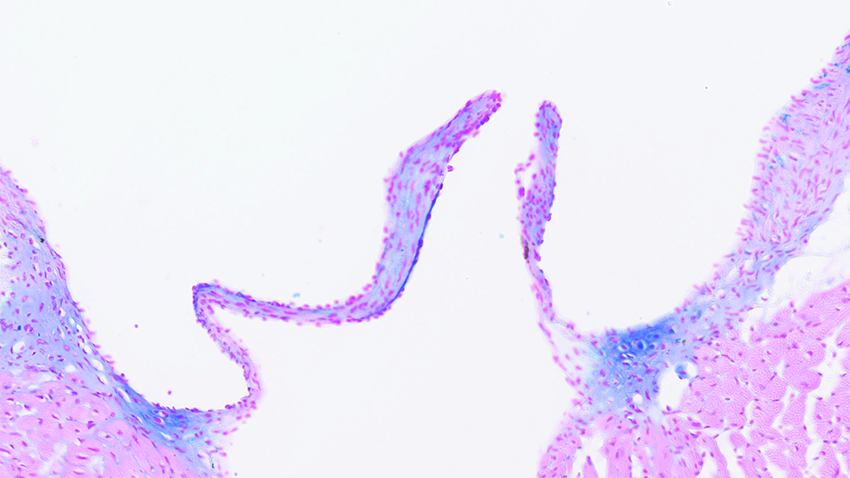

Supplement: Supplementary file 7 — Source data Fig. 4 [file 44321_2025_314_MOESM7_ESM.zip › Figure 4/4A/C1-IDS.IGF2co_AV.tif]

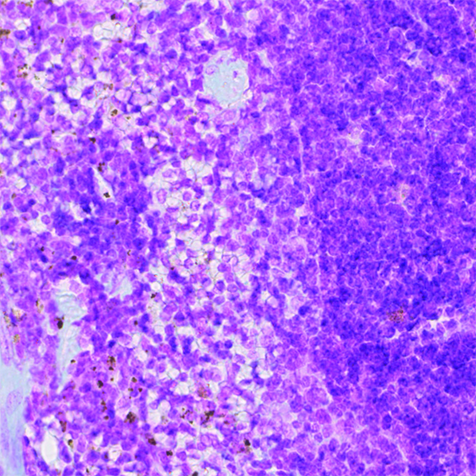

Supplement: Supplementary file 7 — Source data Fig. 4 [file 44321_2025_314_MOESM7_ESM.zip › Figure 4/4A/B1-IDSco_Spleen.tif]

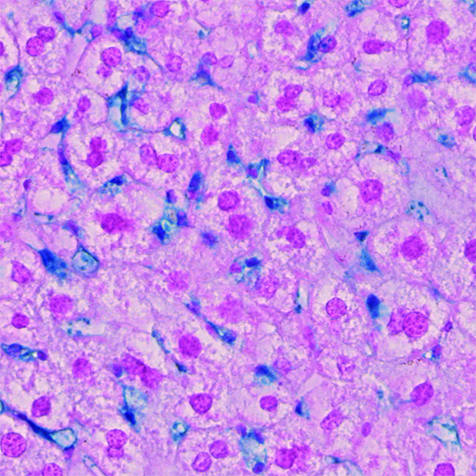

Supplement: Supplementary file 7 — Source data Fig. 4 [file 44321_2025_314_MOESM7_ESM.zip › Figure 4/4A/A7-MPS II.tif]

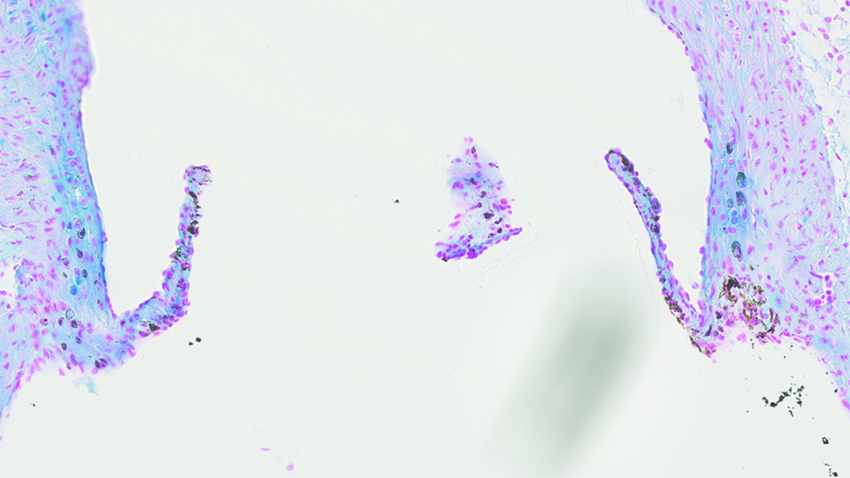

Supplement: Supplementary file 7 — Source data Fig. 4 [file 44321_2025_314_MOESM7_ESM.zip › Figure 4/4A/C3-IDS.IGF2del_co_AV.tif]

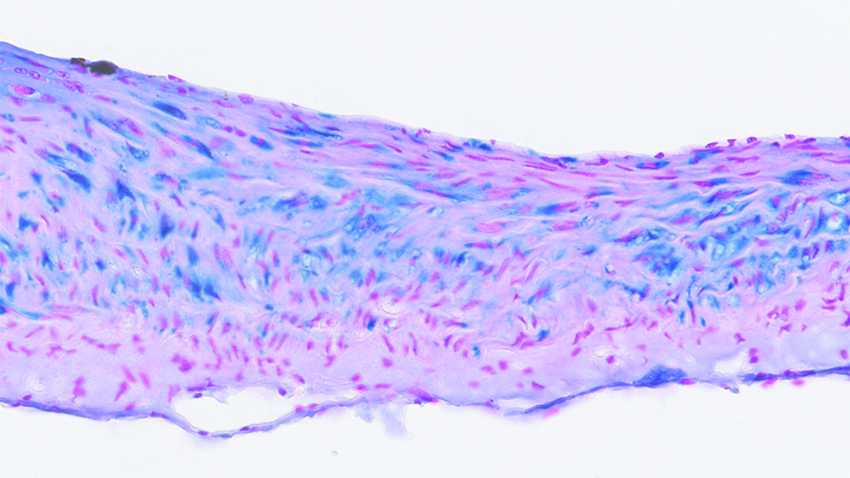

Supplement: Supplementary file 7 — Source data Fig. 4 [file 44321_2025_314_MOESM7_ESM.zip › Figure 4/4A/D1-IDSco.tif]

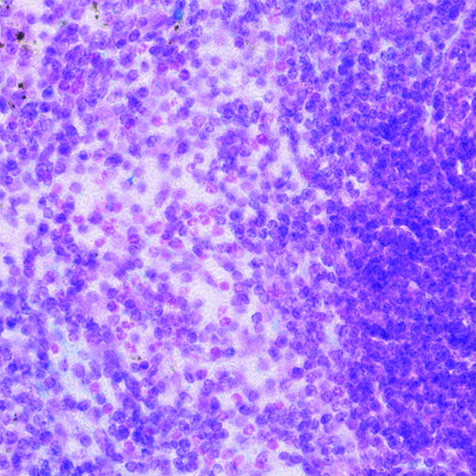

Supplement: Supplementary file 7 — Source data Fig. 4 [file 44321_2025_314_MOESM7_ESM.zip › Figure 4/4A/B4-IDS.SWAP-ApoE2co_Spleen.tif]

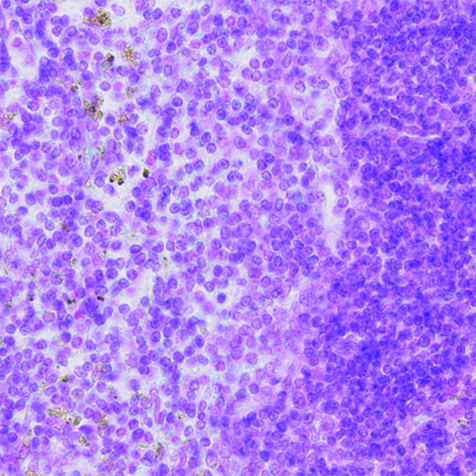

Supplement: Supplementary file 7 — Source data Fig. 4 [file 44321_2025_314_MOESM7_ESM.zip › Figure 4/4A/B3-IDS.IGF2del_co_Spleen.tif]

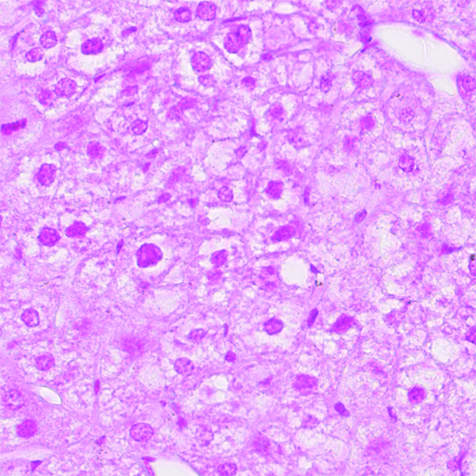

Supplement: Supplementary file 7 — Source data Fig. 4 [file 44321_2025_314_MOESM7_ESM.zip › Figure 4/4A/A1-IDSco_Liver.tif]

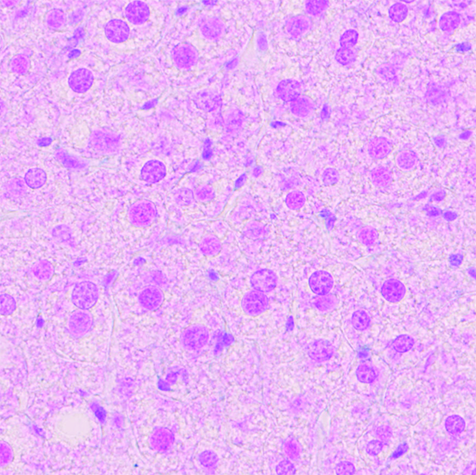

Supplement: Supplementary file 7 — Source data Fig. 4 [file 44321_2025_314_MOESM7_ESM.zip › Figure 4/4A/A5-IDS.SWAP-RAP12x2co_Liver.tif]

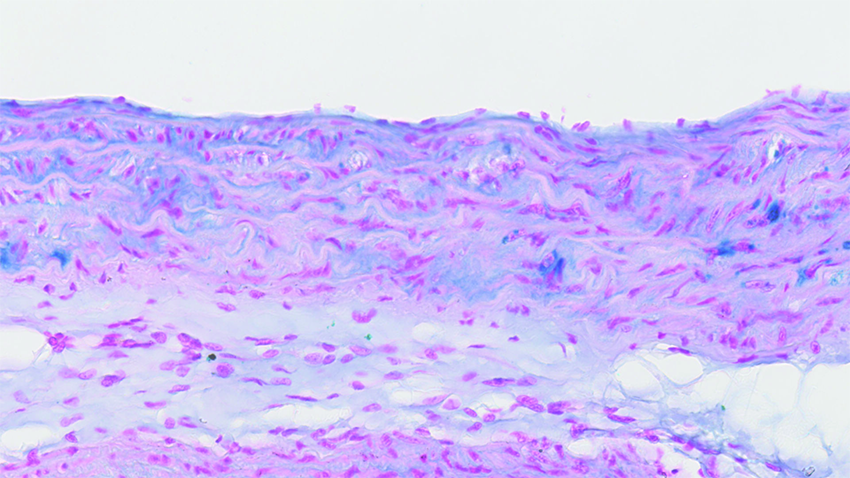

Supplement: Supplementary file 7 — Source data Fig. 4 [file 44321_2025_314_MOESM7_ESM.zip › Figure 4/4A/D2-IDS.IGF2co.tif]

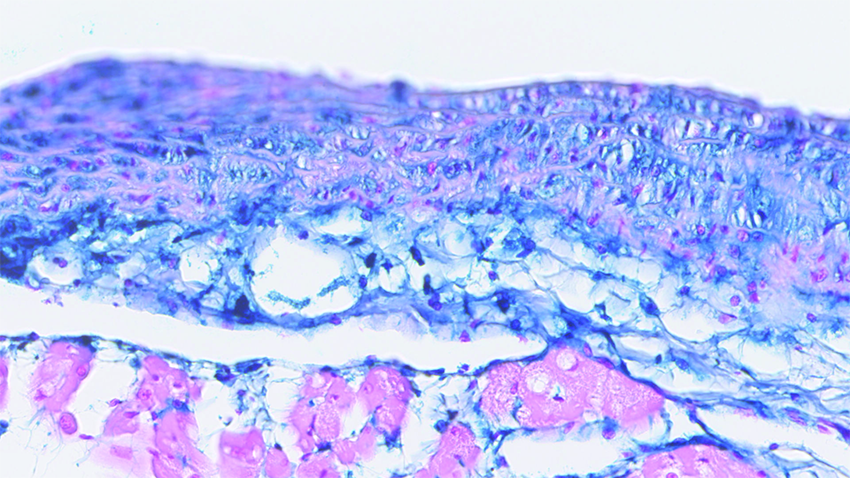

Supplement: Supplementary file 7 — Source data Fig. 4 [file 44321_2025_314_MOESM7_ESM.zip › Figure 4/4A/D7_MPS II.tif]

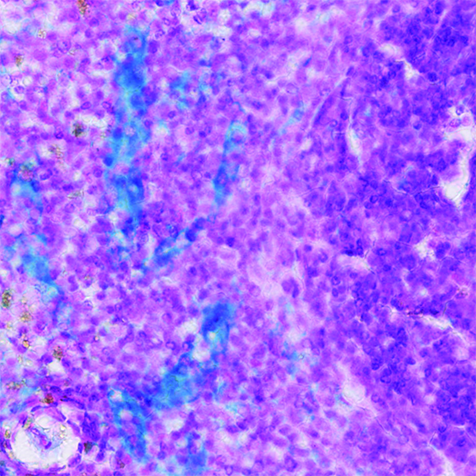

Supplement: Supplementary file 7 — Source data Fig. 4 [file 44321_2025_314_MOESM7_ESM.zip › Figure 4/4A/B6-GFP_Spleen.tif]

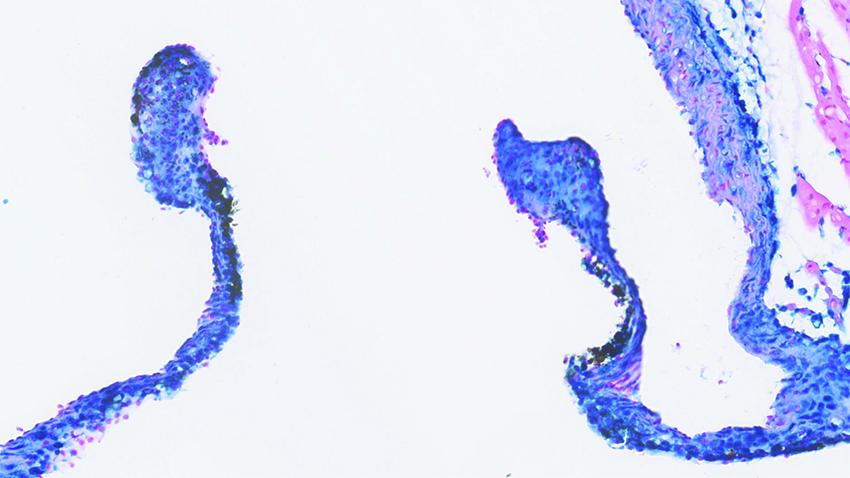

Supplement: Supplementary file 7 — Source data Fig. 4 [file 44321_2025_314_MOESM7_ESM.zip › Figure 4/4A/C7-MPS II_AV.tif]

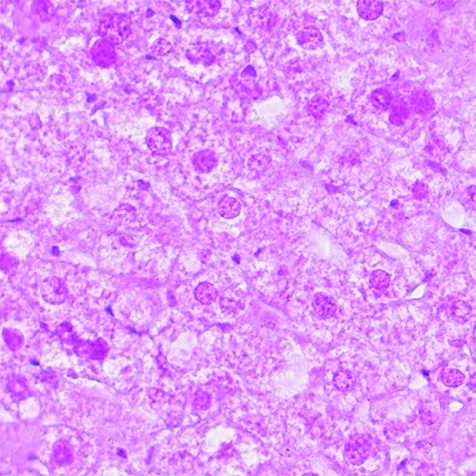

Supplement: Supplementary file 7 — Source data Fig. 4 [file 44321_2025_314_MOESM7_ESM.zip › Figure 4/4A/A2-IDS.IGF2co_Liver.tif]

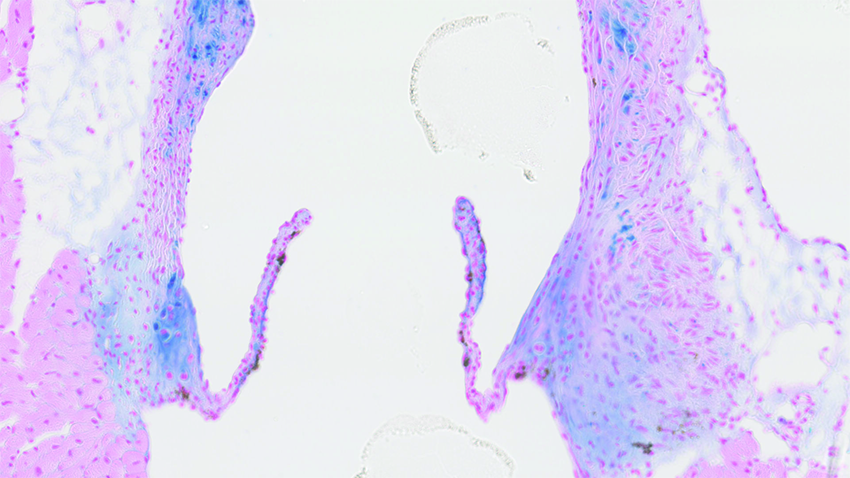

Supplement: Supplementary file 7 — Source data Fig. 4 [file 44321_2025_314_MOESM7_ESM.zip › Figure 4/4A/C1-IDSco_AV.tif]

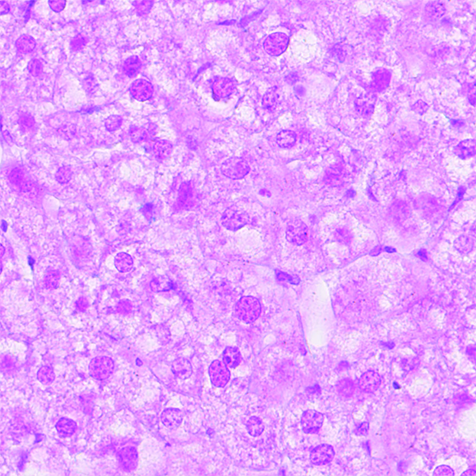

Supplement: Supplementary file 7 — Source data Fig. 4 [file 44321_2025_314_MOESM7_ESM.zip › Figure 4/4A/A4-IDS.SWAP-ApoEco_Liver.tif]

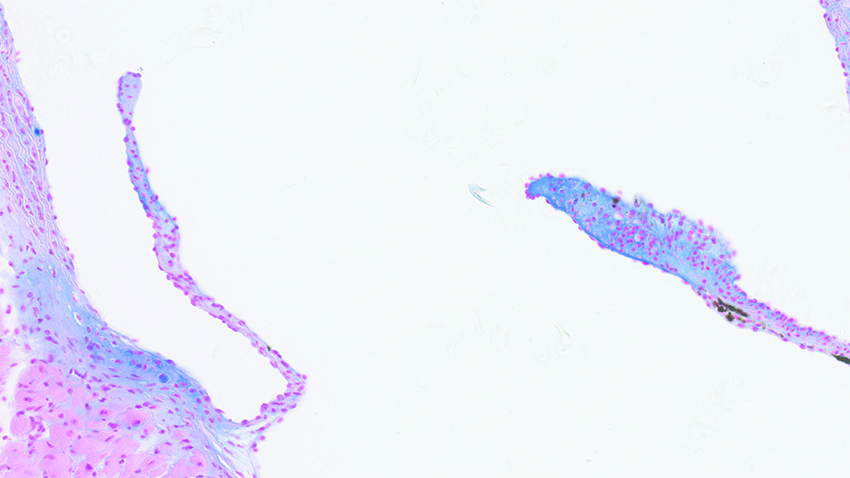

Supplement: Supplementary file 7 — Source data Fig. 4 [file 44321_2025_314_MOESM7_ESM.zip › Figure 4/4A/C4-IDS.SWAP-ApoE2co_AV.tif]

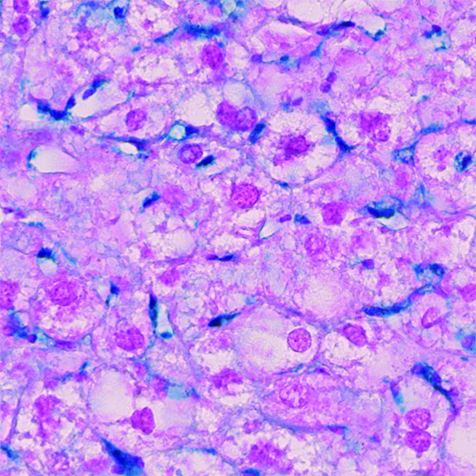

Supplement: Supplementary file 7 — Source data Fig. 4 [file 44321_2025_314_MOESM7_ESM.zip › Figure 4/4A/A6-GFP.tif]

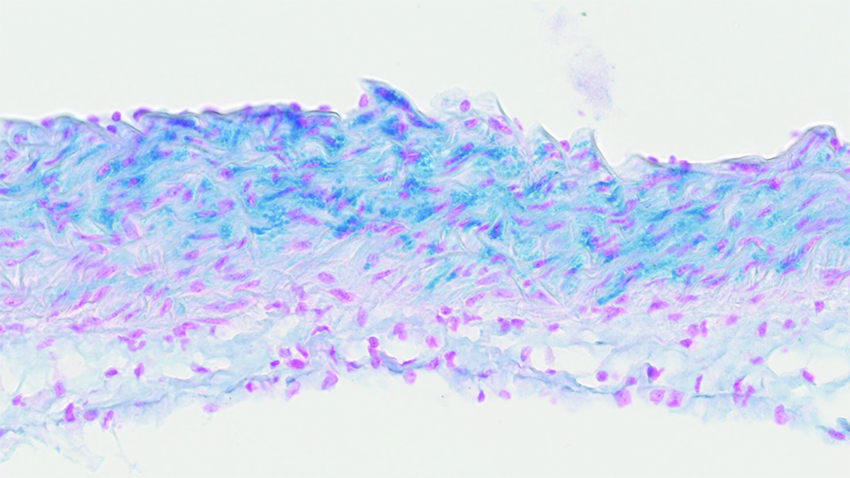

Supplement: Supplementary file 7 — Source data Fig. 4 [file 44321_2025_314_MOESM7_ESM.zip › Figure 4/4A/D3_IDS.IGF2del_co.tif]

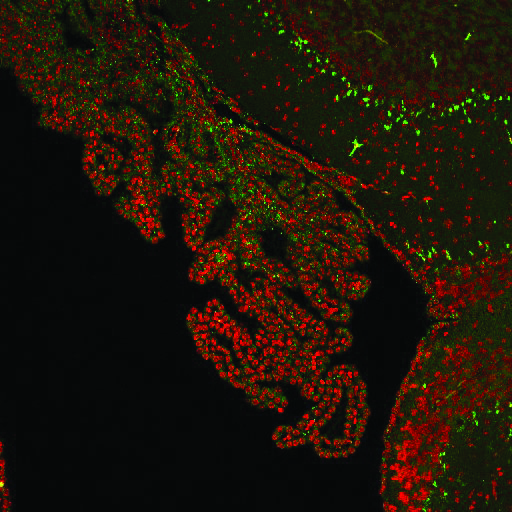

Supplement: Supplementary file 8 — Source data Fig. 5 [file 44321_2025_314_MOESM8_ESM.zip › Figure 5/5E/D5_IDS.SWAP-RAP12x2_co_CP.tif]

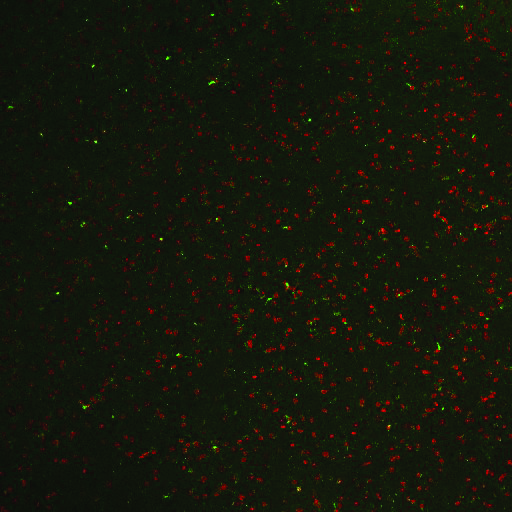

Supplement: Supplementary file 8 — Source data Fig. 5 [file 44321_2025_314_MOESM8_ESM.zip › Figure 5/5E/C7_WT_Thalamus.tif]

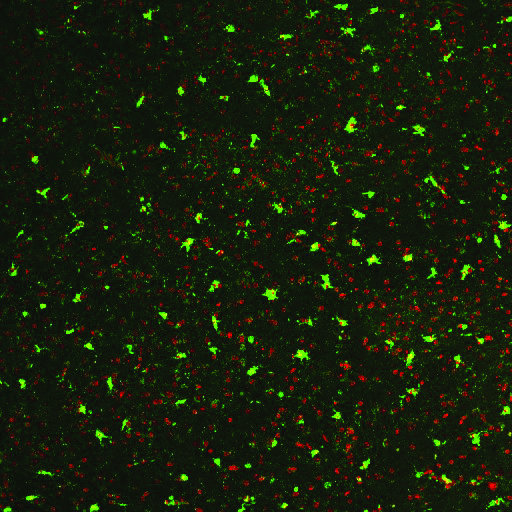

Supplement: Supplementary file 8 — Source data Fig. 5 [file 44321_2025_314_MOESM8_ESM.zip › Figure 5/5E/C1_IDSco_Thalamus.tif]

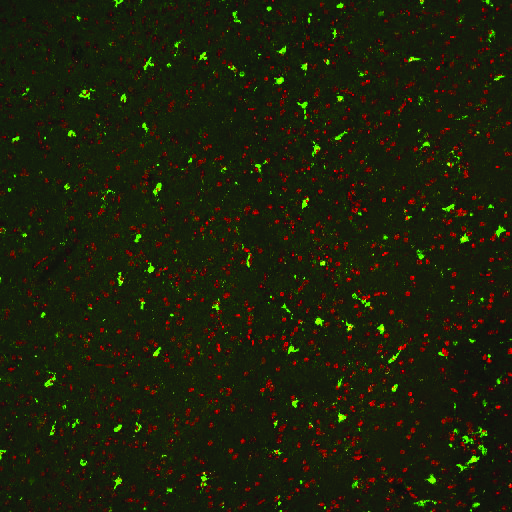

Supplement: Supplementary file 8 — Source data Fig. 5 [file 44321_2025_314_MOESM8_ESM.zip › Figure 5/5E/C3_IDS.IGF2del_co_Thalamus.tif]

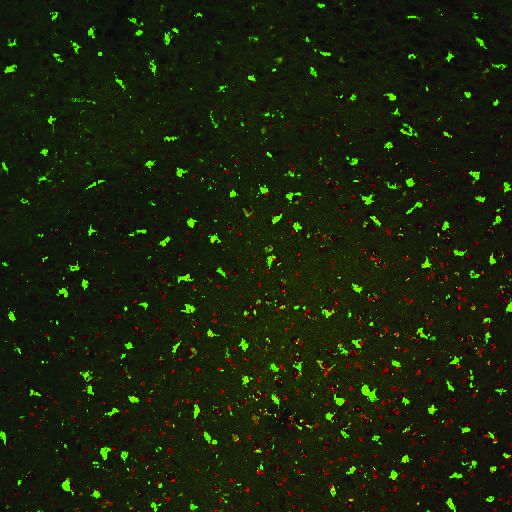

Supplement: Supplementary file 8 — Source data Fig. 5 [file 44321_2025_314_MOESM8_ESM.zip › Figure 5/5E/C6_MPS II_Thalamus.tif]

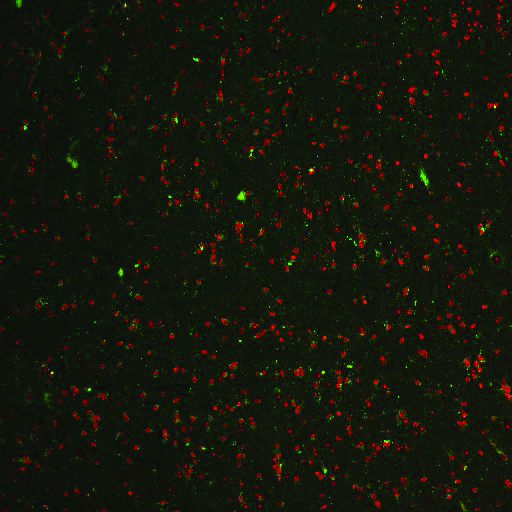

Supplement: Supplementary file 8 — Source data Fig. 5 [file 44321_2025_314_MOESM8_ESM.zip › Figure 5/5E/F7_WT_Brainstem.tif]

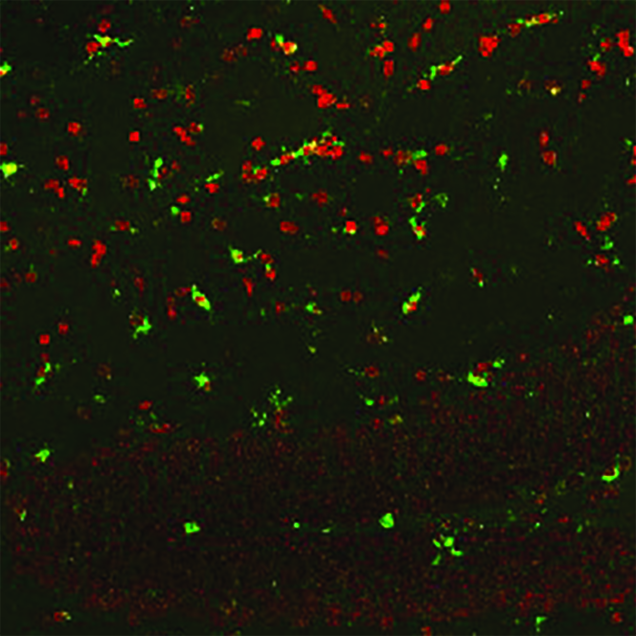

Supplement: Supplementary file 8 — Source data Fig. 5 [file 44321_2025_314_MOESM8_ESM.zip › Figure 5/5E/B3_IDS.IGF2del_co_Hippocampus.tif]

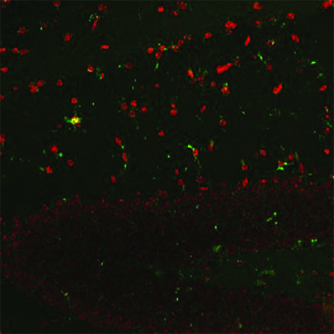

Supplement: Supplementary file 8 — Source data Fig. 5 [file 44321_2025_314_MOESM8_ESM.zip › Figure 5/5E/B2_IDS.IGF2co_Hippocampus.tif]

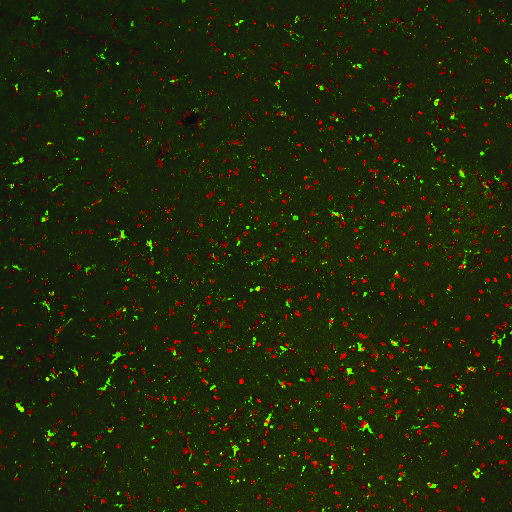

Supplement: Supplementary file 8 — Source data Fig. 5 [file 44321_2025_314_MOESM8_ESM.zip › Figure 5/5E/C5_IDS.SWAP-RAP12x2_Thalamus.tif]

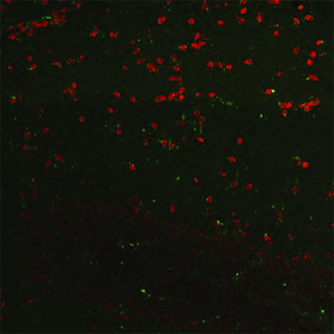

Supplement: Supplementary file 8 — Source data Fig. 5 [file 44321_2025_314_MOESM8_ESM.zip › Figure 5/5E/B7_WT_Hippocampus.tif]

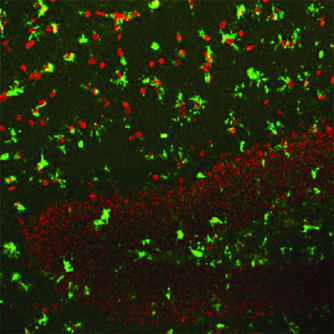

Supplement: Supplementary file 8 — Source data Fig. 5 [file 44321_2025_314_MOESM8_ESM.zip › Figure 5/5E/B6_MPS II_Hippocampus.tif]

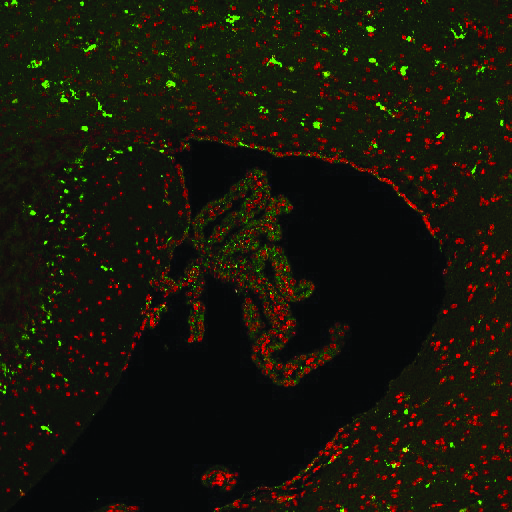

Supplement: Supplementary file 8 — Source data Fig. 5 [file 44321_2025_314_MOESM8_ESM.zip › Figure 5/5E/D3_IDS.IGF2del_co_CP.tif]

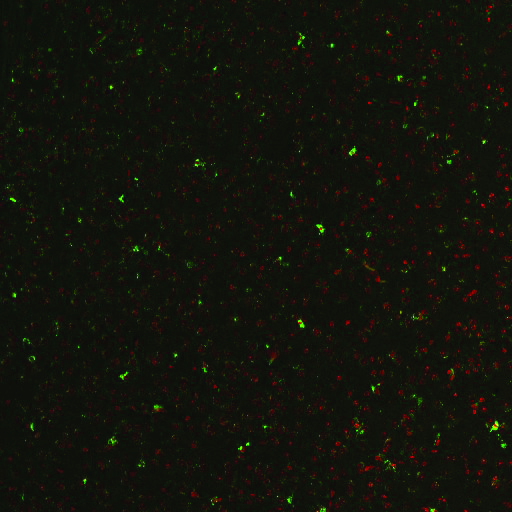

Supplement: Supplementary file 8 — Source data Fig. 5 [file 44321_2025_314_MOESM8_ESM.zip › Figure 5/5E/A2_IDS.IGF2co_Cortex.tif]

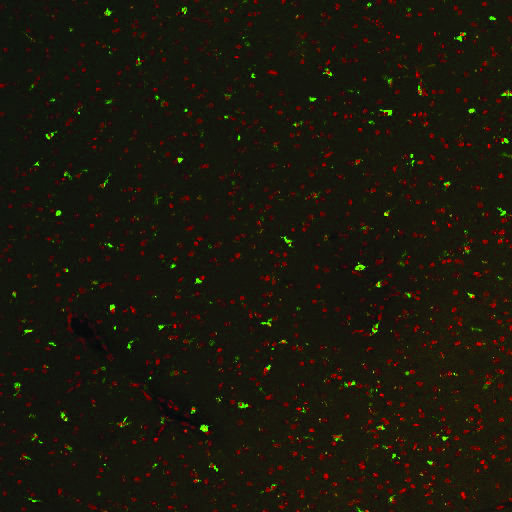

Supplement: Supplementary file 8 — Source data Fig. 5 [file 44321_2025_314_MOESM8_ESM.zip › Figure 5/5E/C4_IDS.SWAP-ApoEco_Thalamus.tif]

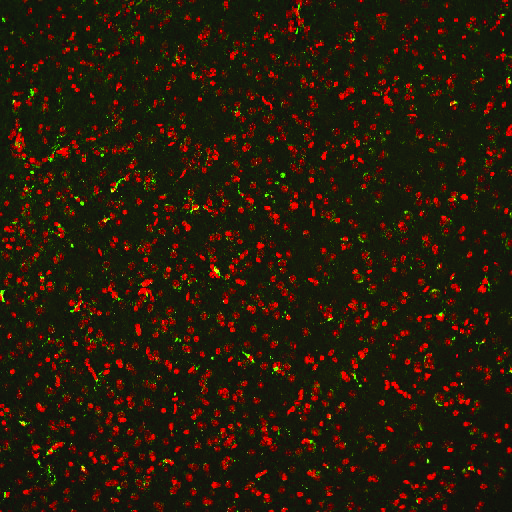

Supplement: Supplementary file 8 — Source data Fig. 5 [file 44321_2025_314_MOESM8_ESM.zip › Figure 5/5E/A5_IDS.SWAP-RAP12x2co_Cortex.tif]

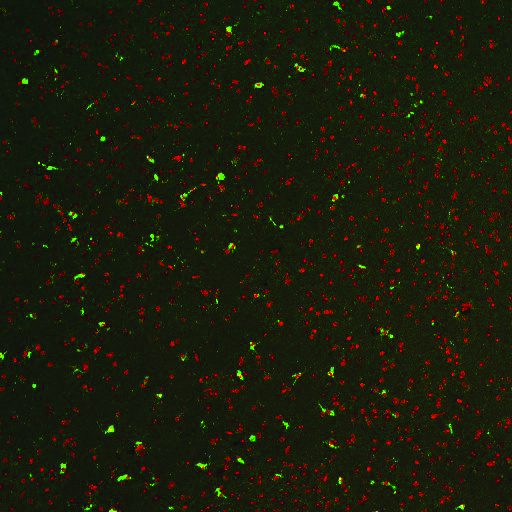

Supplement: Supplementary file 8 — Source data Fig. 5 [file 44321_2025_314_MOESM8_ESM.zip › Figure 5/5E/C2_IDS.IGF2co_Thalamus.tif]

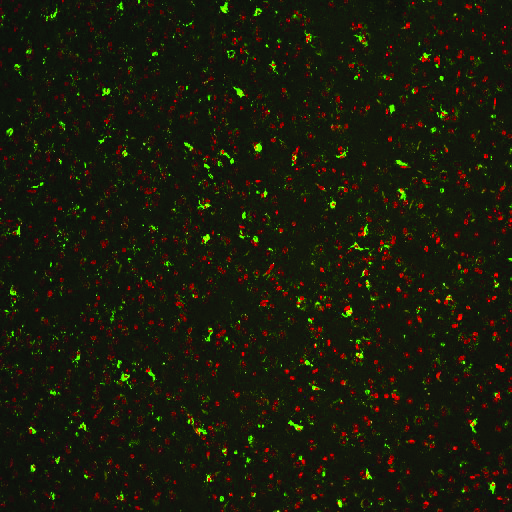

Supplement: Supplementary file 8 — Source data Fig. 5 [file 44321_2025_314_MOESM8_ESM.zip › Figure 5/5E/A1_IDSco_Cortex.tif]

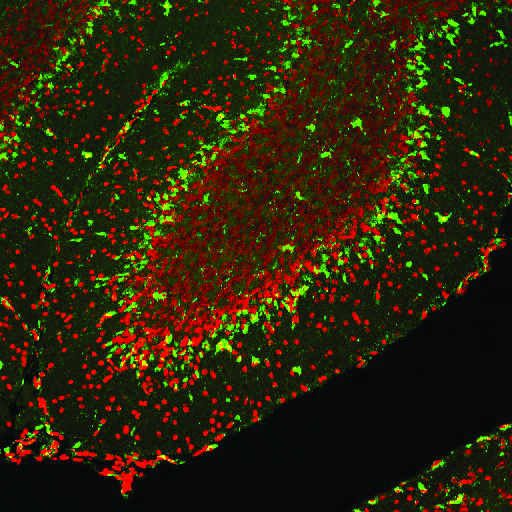

Supplement: Supplementary file 8 — Source data Fig. 5 [file 44321_2025_314_MOESM8_ESM.zip › Figure 5/5E/E6_MPS II_Cerebellum.tif]

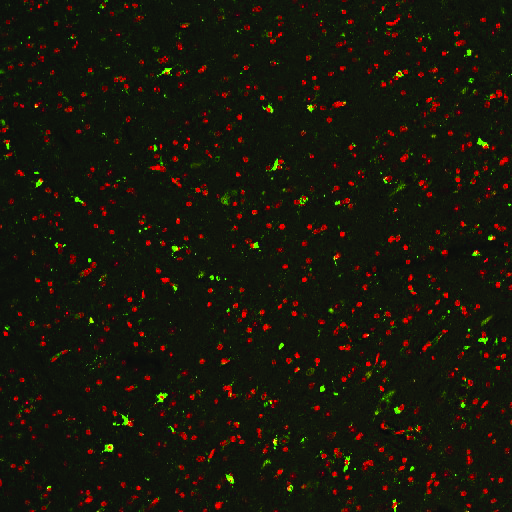

Supplement: Supplementary file 8 — Source data Fig. 5 [file 44321_2025_314_MOESM8_ESM.zip › Figure 5/5E/F1_IDSco_Brainstem.tif]

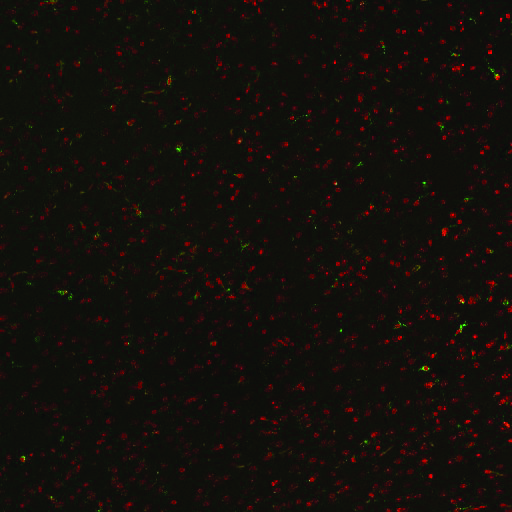

Supplement: Supplementary file 8 — Source data Fig. 5 [file 44321_2025_314_MOESM8_ESM.zip › Figure 5/5E/A4_IDS.SWAP-ApoE2co_Cortex.tif]

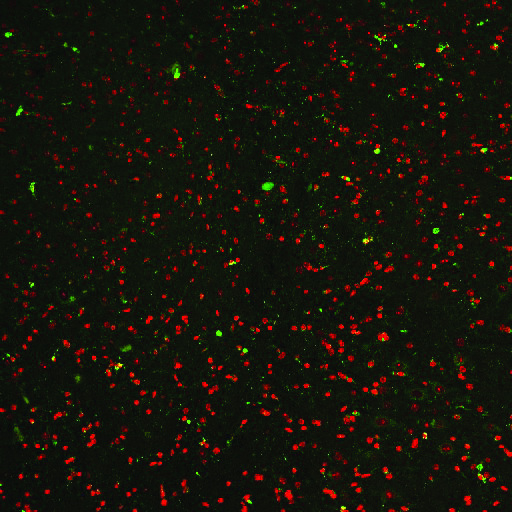

Supplement: Supplementary file 8 — Source data Fig. 5 [file 44321_2025_314_MOESM8_ESM.zip › Figure 5/5E/F2_IDS.IGF2co_Brainstem.tif]

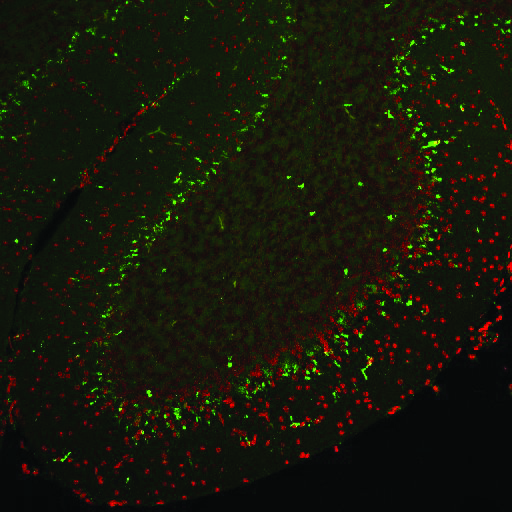

Supplement: Supplementary file 8 — Source data Fig. 5 [file 44321_2025_314_MOESM8_ESM.zip › Figure 5/5E/E4_IDS.SWAP-ApoE2co_Cerebellum.tif]

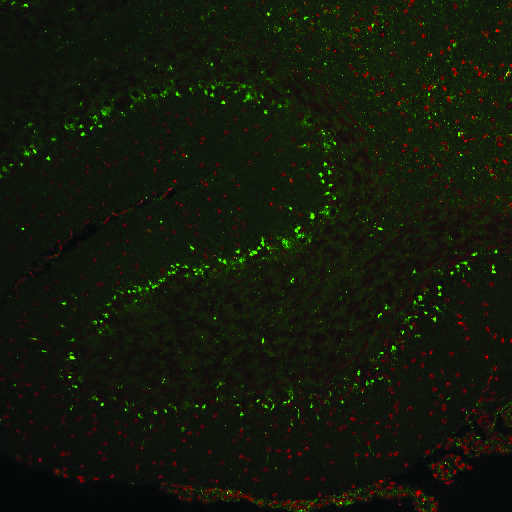

Supplement: Supplementary file 8 — Source data Fig. 5 [file 44321_2025_314_MOESM8_ESM.zip › Figure 5/5E/E7_WT_Cerebellum.tif]

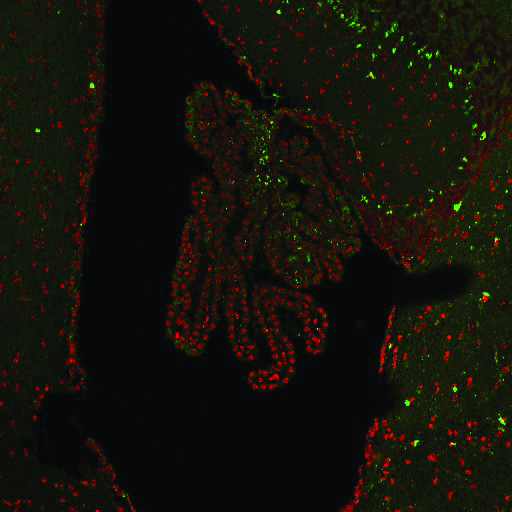

Supplement: Supplementary file 8 — Source data Fig. 5 [file 44321_2025_314_MOESM8_ESM.zip › Figure 5/5E/D2_IDS.IGF2co_CP.tif]

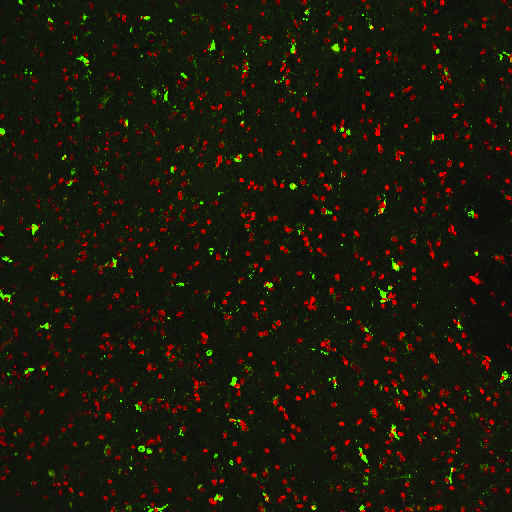

Supplement: Supplementary file 8 — Source data Fig. 5 [file 44321_2025_314_MOESM8_ESM.zip › Figure 5/5E/F3_IDS.IGF2del_co_Brainstem.tif]

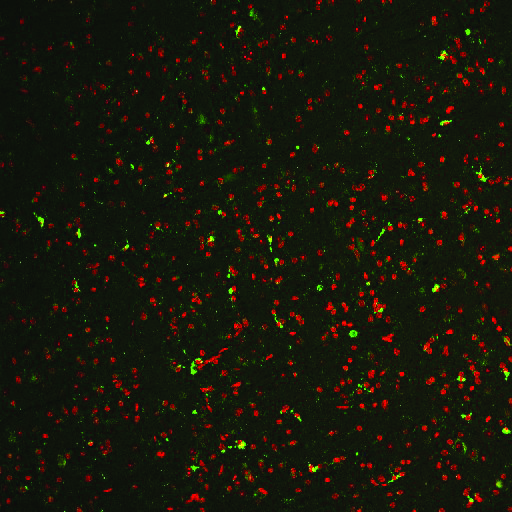

Supplement: Supplementary file 8 — Source data Fig. 5 [file 44321_2025_314_MOESM8_ESM.zip › Figure 5/5E/F4_IDS.SWAP-ApoE2co_Brainstem.tif]

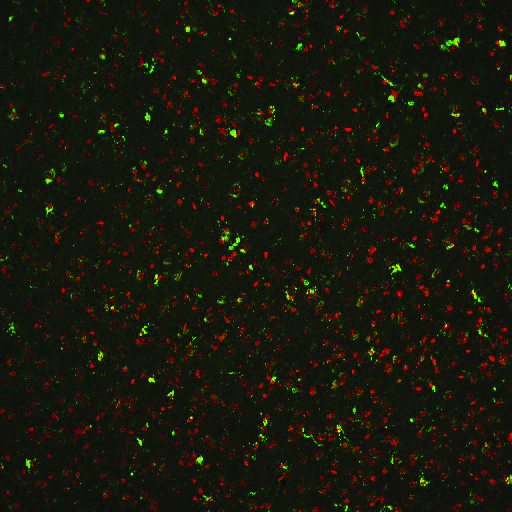

Supplement: Supplementary file 8 — Source data Fig. 5 [file 44321_2025_314_MOESM8_ESM.zip › Figure 5/5E/A3_IDS.IGF2del_co_Cortex.tif]

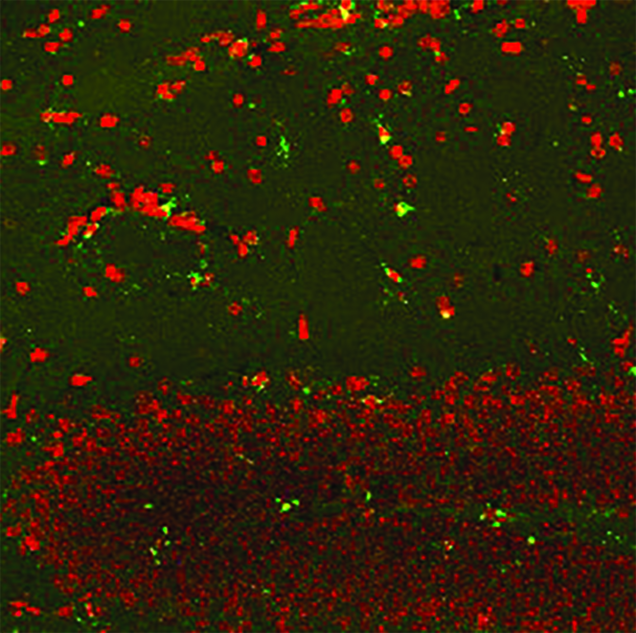

Supplement: Supplementary file 8 — Source data Fig. 5 [file 44321_2025_314_MOESM8_ESM.zip › Figure 5/5E/B5_IDS.SWAP-RAP12x2co_Hippocampus.tif]

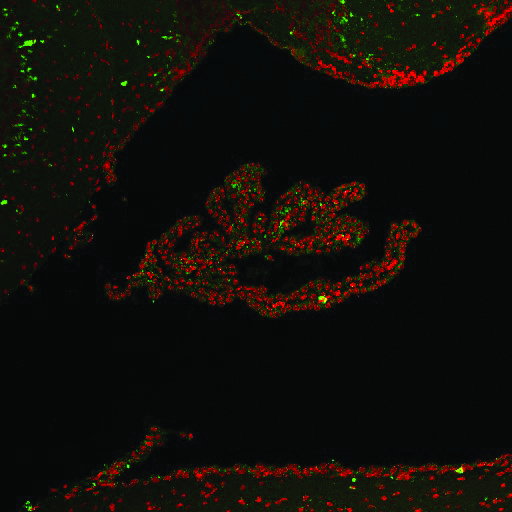

Supplement: Supplementary file 8 — Source data Fig. 5 [file 44321_2025_314_MOESM8_ESM.zip › Figure 5/5E/D4_IDS.SWAP-ApoE_co_CP.tif]

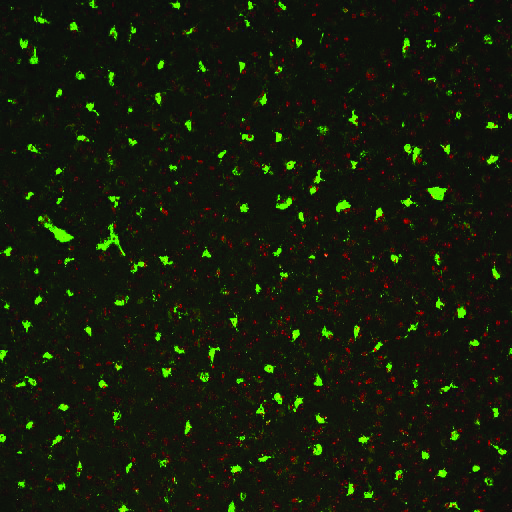

Supplement: Supplementary file 8 — Source data Fig. 5 [file 44321_2025_314_MOESM8_ESM.zip › Figure 5/5E/A6_MPS II_Cortex.tif]

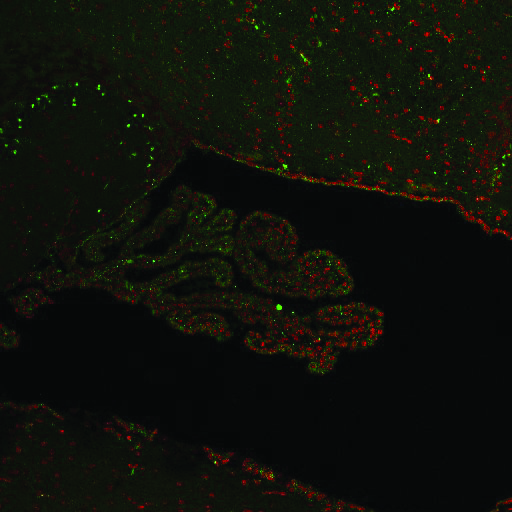

Supplement: Supplementary file 8 — Source data Fig. 5 [file 44321_2025_314_MOESM8_ESM.zip › Figure 5/5E/D7_WT_CP.tif]

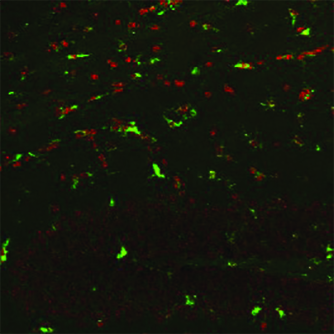

Supplement: Supplementary file 8 — Source data Fig. 5 [file 44321_2025_314_MOESM8_ESM.zip › Figure 5/5E/B1_IDSco_Hippocampus.tif]

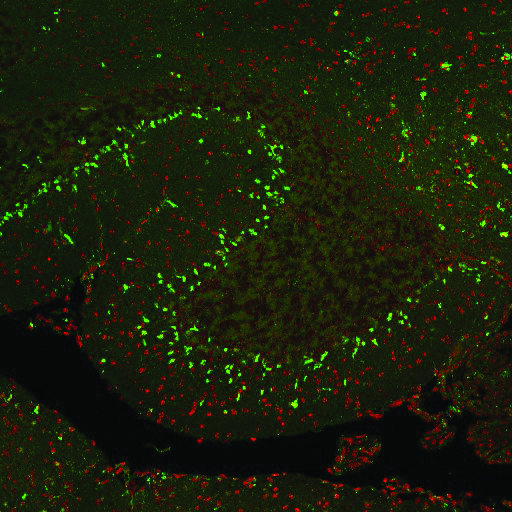

Supplement: Supplementary file 8 — Source data Fig. 5 [file 44321_2025_314_MOESM8_ESM.zip › Figure 5/5E/E5_IDS.SWAP-RAP12x2co_Cerebellum.tif]

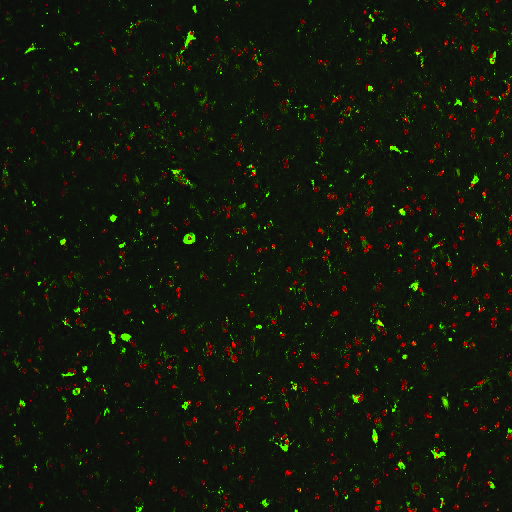

Supplement: Supplementary file 8 — Source data Fig. 5 [file 44321_2025_314_MOESM8_ESM.zip › Figure 5/5E/F6_MPS II_Brainstem.tif]

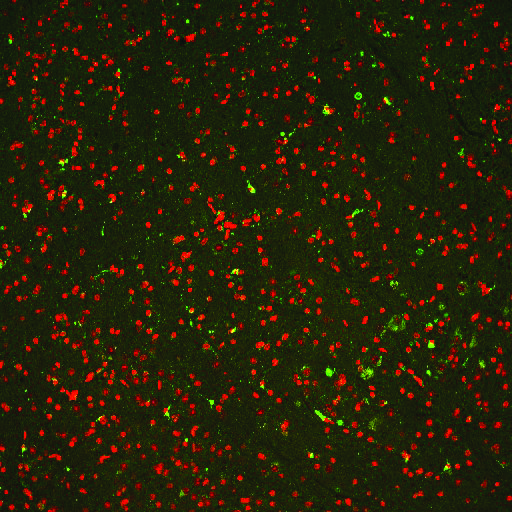

Supplement: Supplementary file 8 — Source data Fig. 5 [file 44321_2025_314_MOESM8_ESM.zip › Figure 5/5E/F5_IDS.SWAP-RAP12x2co_Brainstem.tif]

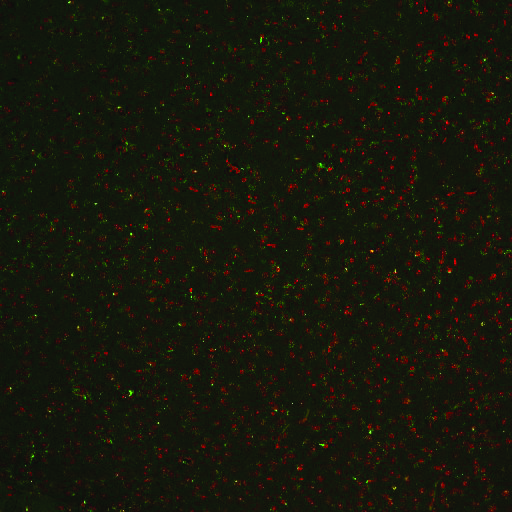

Supplement: Supplementary file 8 — Source data Fig. 5 [file 44321_2025_314_MOESM8_ESM.zip › Figure 5/5E/A7_WT_Cortex.tif]

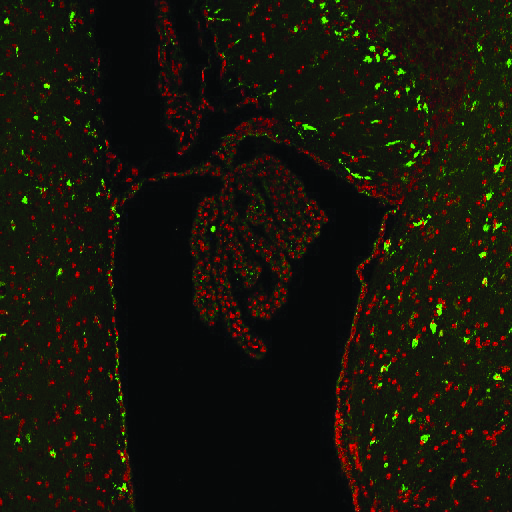

Supplement: Supplementary file 8 — Source data Fig. 5 [file 44321_2025_314_MOESM8_ESM.zip › Figure 5/5E/D1_IDSco_CP.tif]

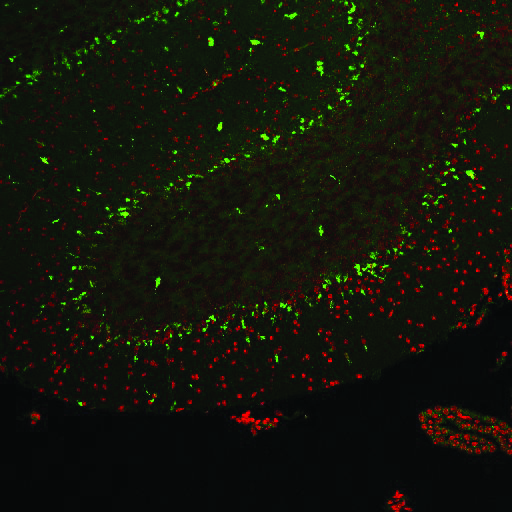

Supplement: Supplementary file 8 — Source data Fig. 5 [file 44321_2025_314_MOESM8_ESM.zip › Figure 5/5E/E3_IDS.IGF2del_co_Cerebellum.tif]

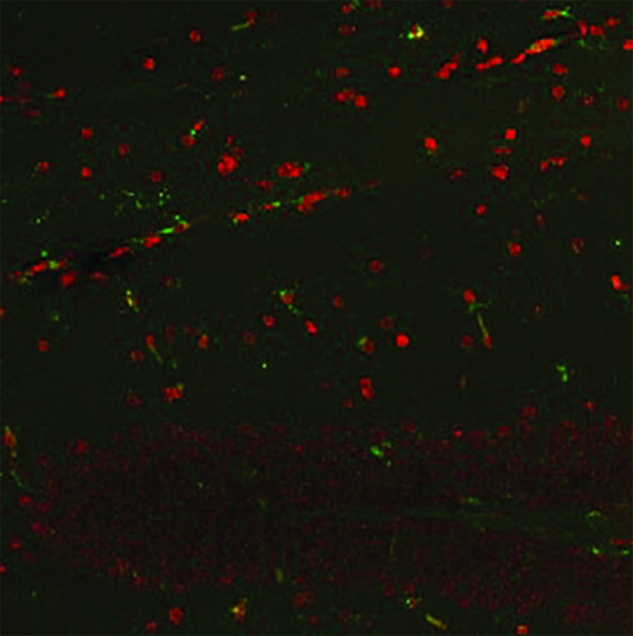

Supplement: Supplementary file 8 — Source data Fig. 5 [file 44321_2025_314_MOESM8_ESM.zip › Figure 5/5E/B4_IDS.SWAP-ApoE2co_Hippocampus.tif]

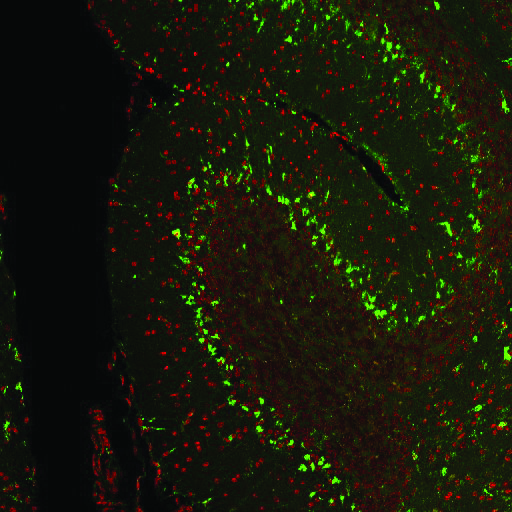

Supplement: Supplementary file 8 — Source data Fig. 5 [file 44321_2025_314_MOESM8_ESM.zip › Figure 5/5E/E1_IDSco_Cerebellum.tif]

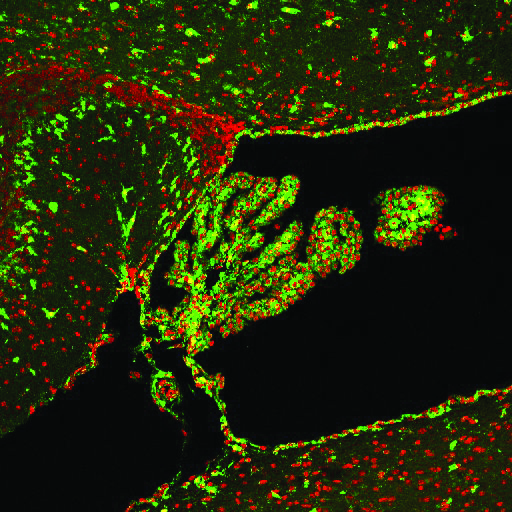

Supplement: Supplementary file 8 — Source data Fig. 5 [file 44321_2025_314_MOESM8_ESM.zip › Figure 5/5E/D6_MPS II_CP.tif]

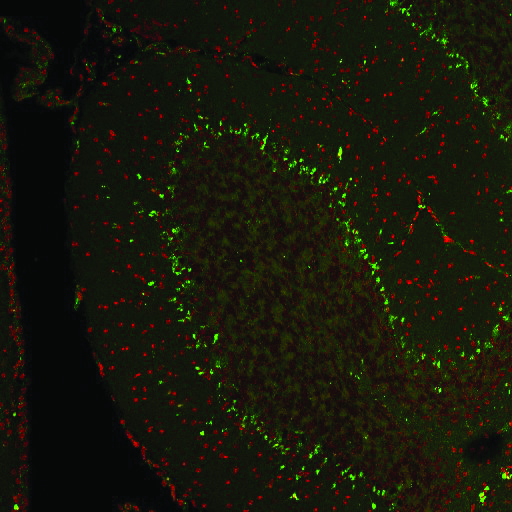

Supplement: Supplementary file 8 — Source data Fig. 5 [file 44321_2025_314_MOESM8_ESM.zip › Figure 5/5E/E2_IDS.IGF2co_Cerebellum.tif]

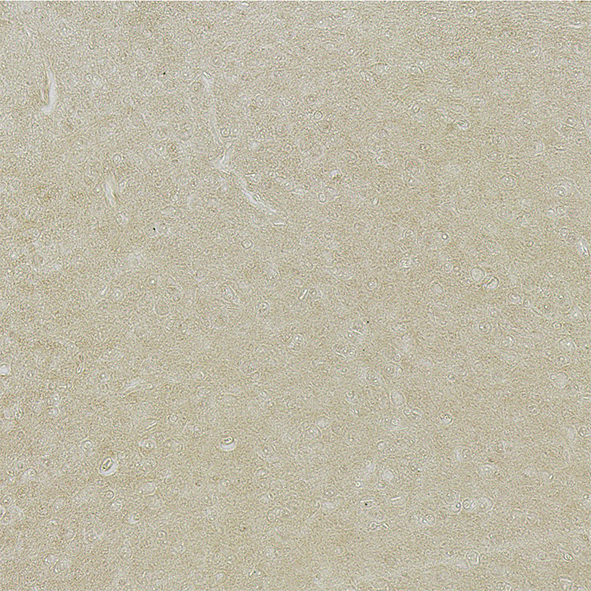

Supplement: Supplementary file 9 — Source data Fig. 6 [file 44321_2025_314_MOESM9_ESM.zip › Figure 6/C7_WT_Thalamus.tif]

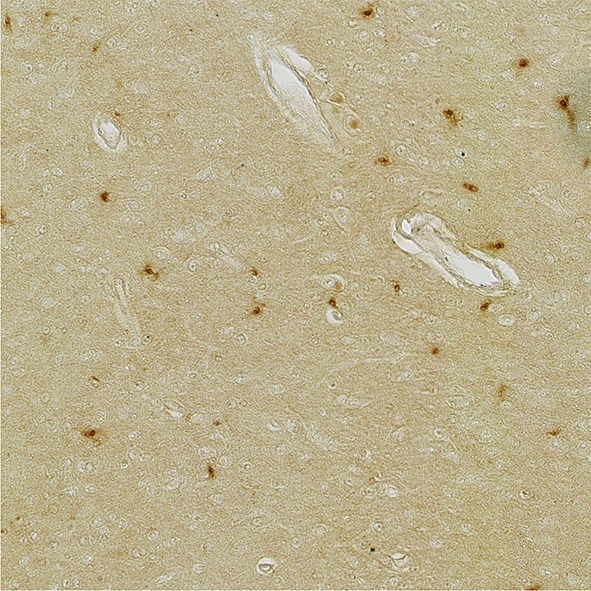

Supplement: Supplementary file 9 — Source data Fig. 6 [file 44321_2025_314_MOESM9_ESM.zip › Figure 6/C1_IDSco_Thalamus.tif]

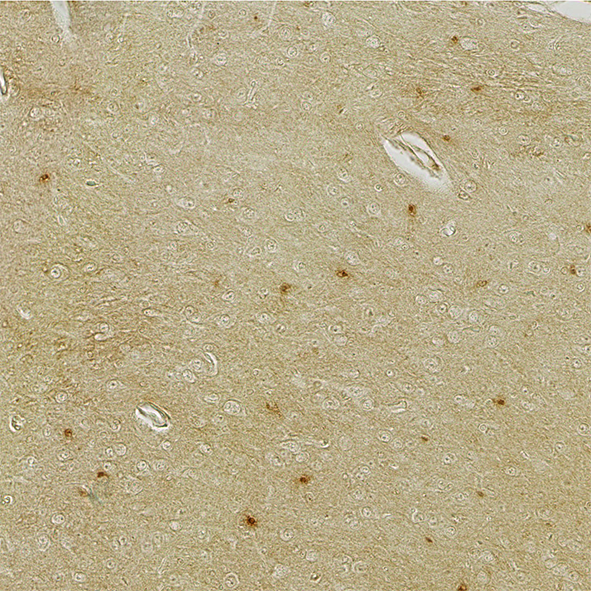

Supplement: Supplementary file 9 — Source data Fig. 6 [file 44321_2025_314_MOESM9_ESM.zip › Figure 6/C3_IDS.IGF2del_co_Thalamus.tif]

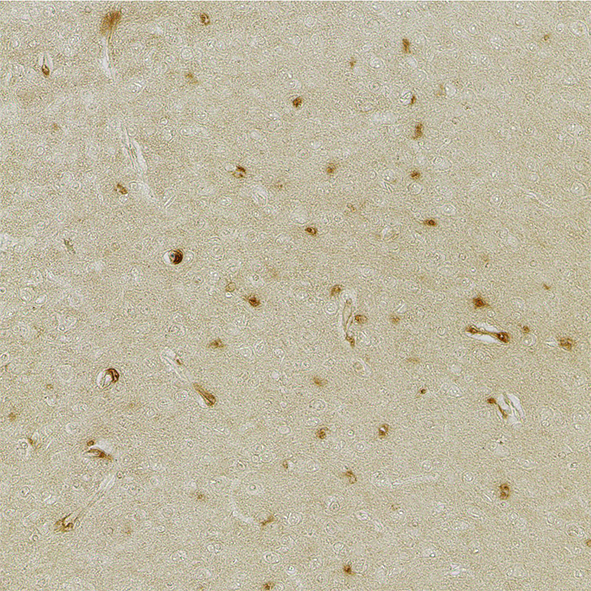

Supplement: Supplementary file 9 — Source data Fig. 6 [file 44321_2025_314_MOESM9_ESM.zip › Figure 6/C6_MPS II_Thalamus.tif]

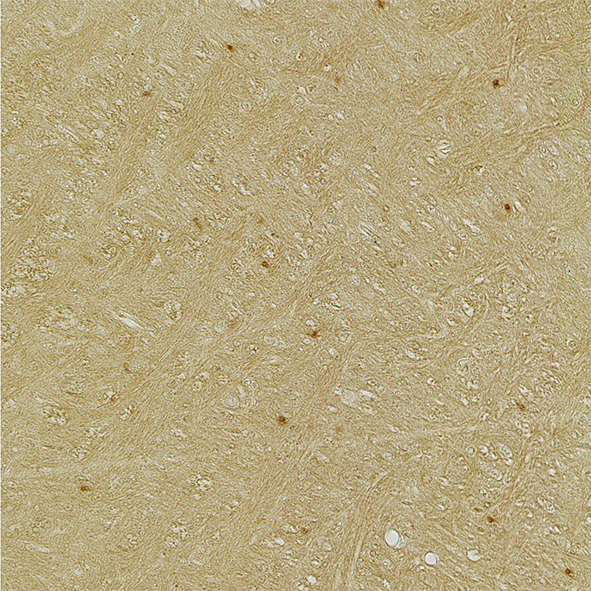

Supplement: Supplementary file 9 — Source data Fig. 6 [file 44321_2025_314_MOESM9_ESM.zip › Figure 6/D1_IDSco_Midbrain.tif]

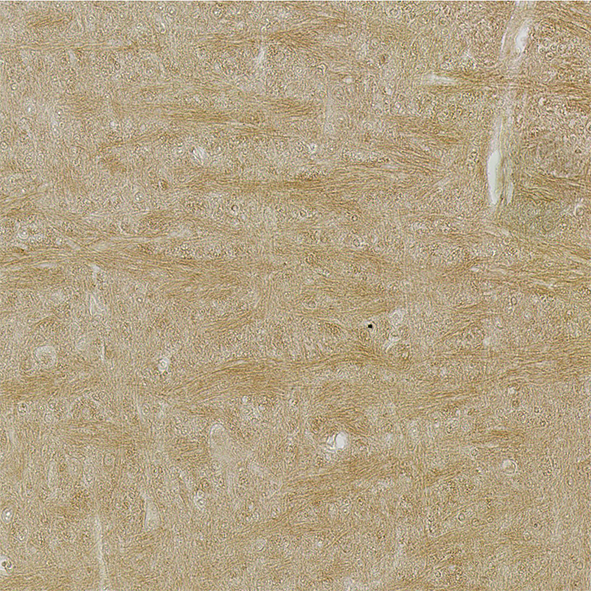

Supplement: Supplementary file 9 — Source data Fig. 6 [file 44321_2025_314_MOESM9_ESM.zip › Figure 6/F7_WT_Brainstem.tif]

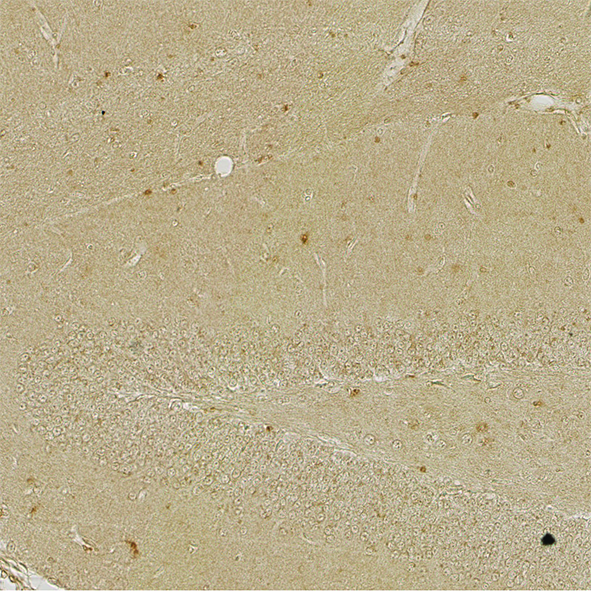

Supplement: Supplementary file 9 — Source data Fig. 6 [file 44321_2025_314_MOESM9_ESM.zip › Figure 6/B3_IDS.IGF2del_co_Hippocampus.tif]

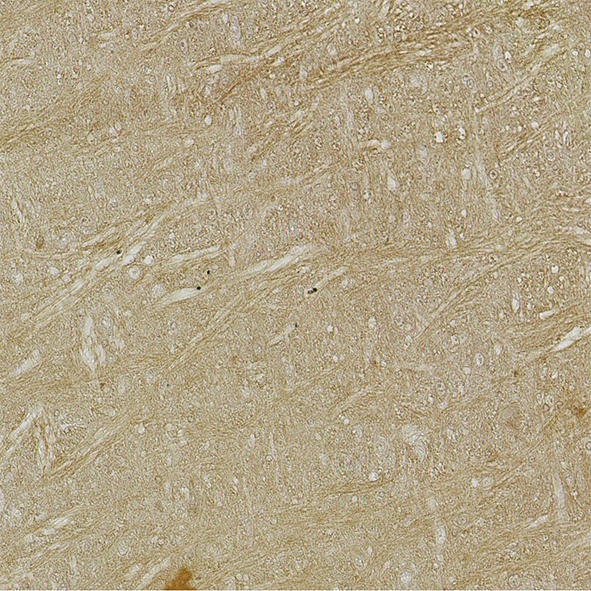

Supplement: Supplementary file 9 — Source data Fig. 6 [file 44321_2025_314_MOESM9_ESM.zip › Figure 6/F5_IDS.SWAP-RAP12x2_Brainstem.tif]

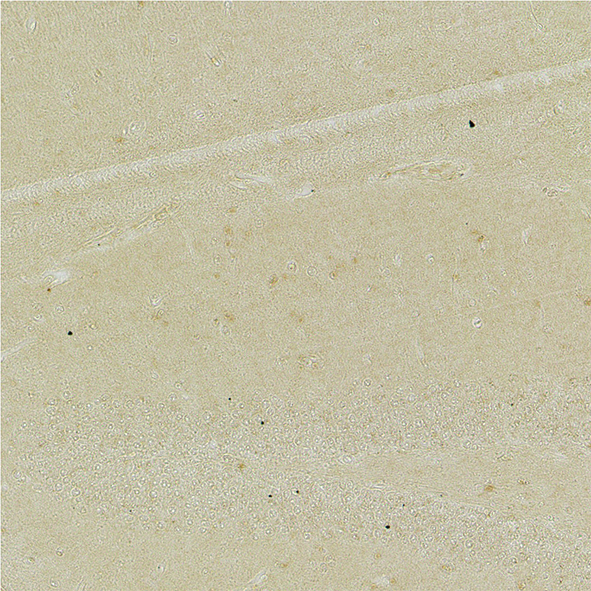

Supplement: Supplementary file 9 — Source data Fig. 6 [file 44321_2025_314_MOESM9_ESM.zip › Figure 6/B2_IDS.IGF2co_Hippocampus.tif]

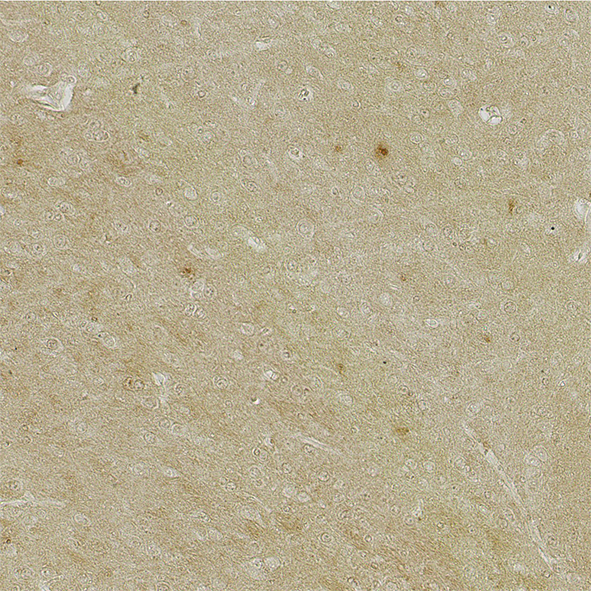

Supplement: Supplementary file 9 — Source data Fig. 6 [file 44321_2025_314_MOESM9_ESM.zip › Figure 6/C5_IDS.SWAP-RAP12x2_Thalamus.tif]

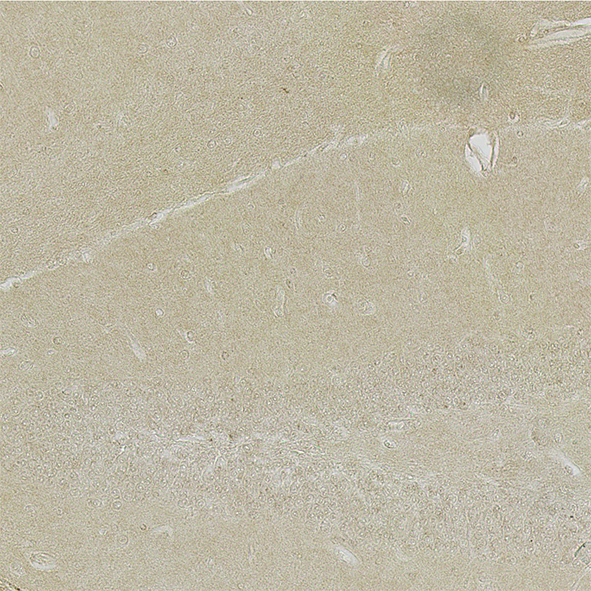

Supplement: Supplementary file 9 — Source data Fig. 6 [file 44321_2025_314_MOESM9_ESM.zip › Figure 6/B7_WT_Hippocampus.tif]

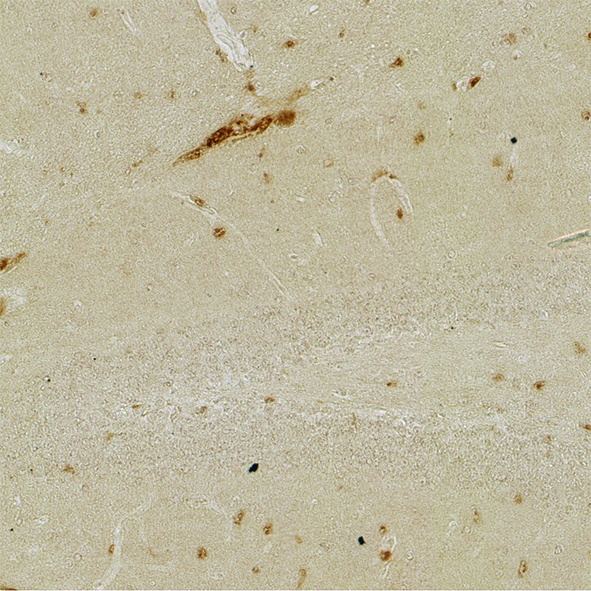

Supplement: Supplementary file 9 — Source data Fig. 6 [file 44321_2025_314_MOESM9_ESM.zip › Figure 6/B6_MPS II_Hippocampus.tif]

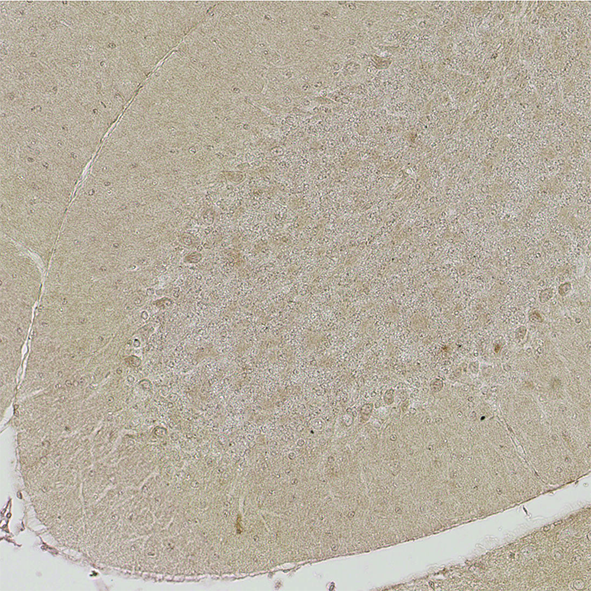

Supplement: Supplementary file 9 — Source data Fig. 6 [file 44321_2025_314_MOESM9_ESM.zip › Figure 6/E5_IDS.SWAP-RAP12x2_Cerebellum.tif]

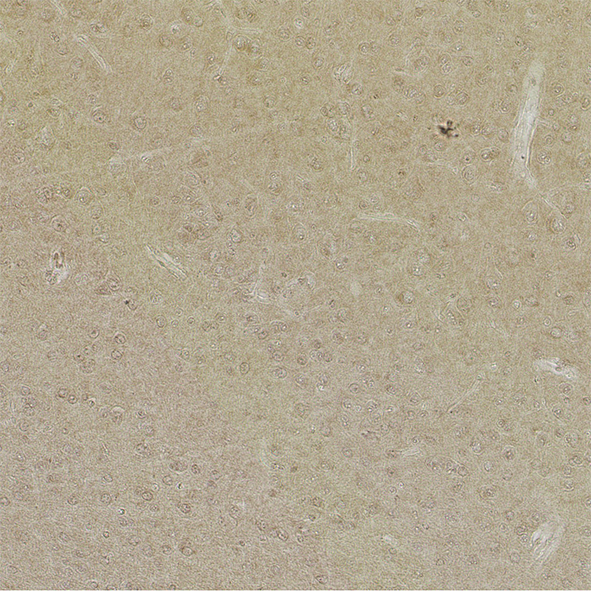

Supplement: Supplementary file 9 — Source data Fig. 6 [file 44321_2025_314_MOESM9_ESM.zip › Figure 6/A5_IDS.SWAP-RAP12x2_Cortex.tif]

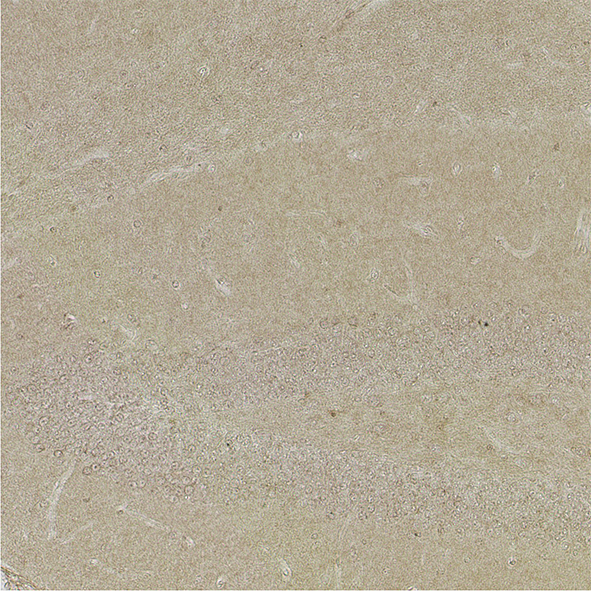

Supplement: Supplementary file 9 — Source data Fig. 6 [file 44321_2025_314_MOESM9_ESM.zip › Figure 6/B5_IDS.SWAP-RAP12x2_Hippocampus.tif]

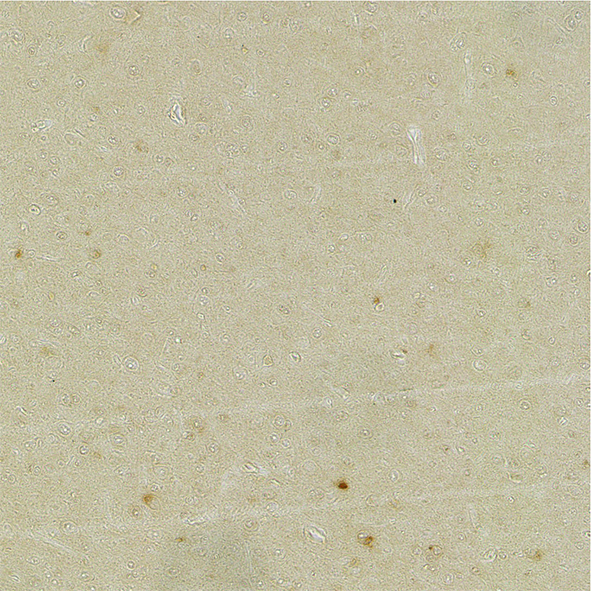

Supplement: Supplementary file 9 — Source data Fig. 6 [file 44321_2025_314_MOESM9_ESM.zip › Figure 6/A2_IDS.IGF2co_Cortex.tif]

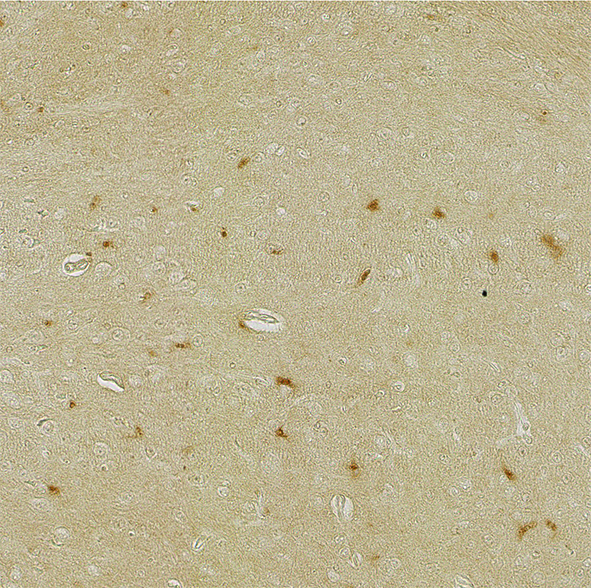

Supplement: Supplementary file 9 — Source data Fig. 6 [file 44321_2025_314_MOESM9_ESM.zip › Figure 6/C4_IDS.SWAP-ApoEco_Thalamus.tif]

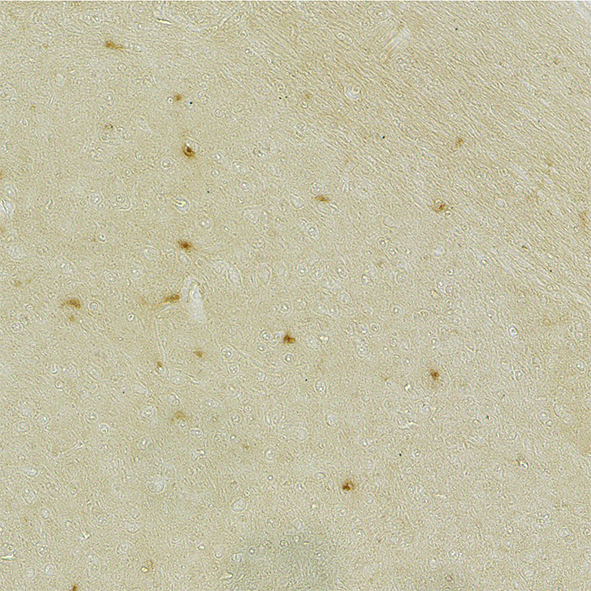

Supplement: Supplementary file 9 — Source data Fig. 6 [file 44321_2025_314_MOESM9_ESM.zip › Figure 6/C2_IDS.IGF2co_Thalamus.tif]

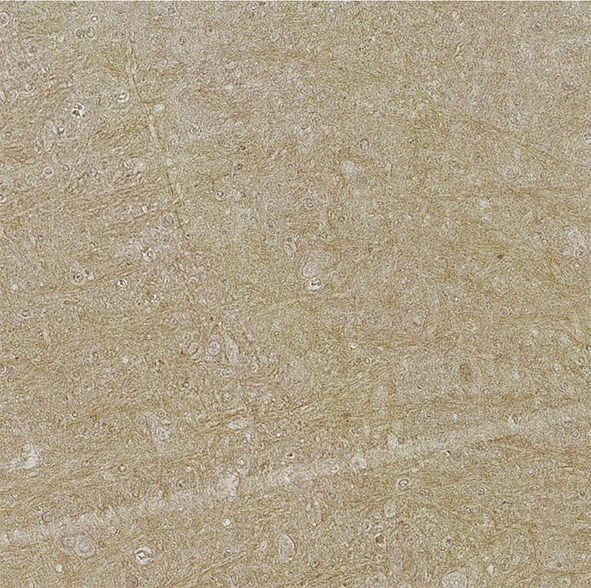

Supplement: Supplementary file 9 — Source data Fig. 6 [file 44321_2025_314_MOESM9_ESM.zip › Figure 6/D5_IDS.SWAP-RAP12x2_Midbrain.tif]

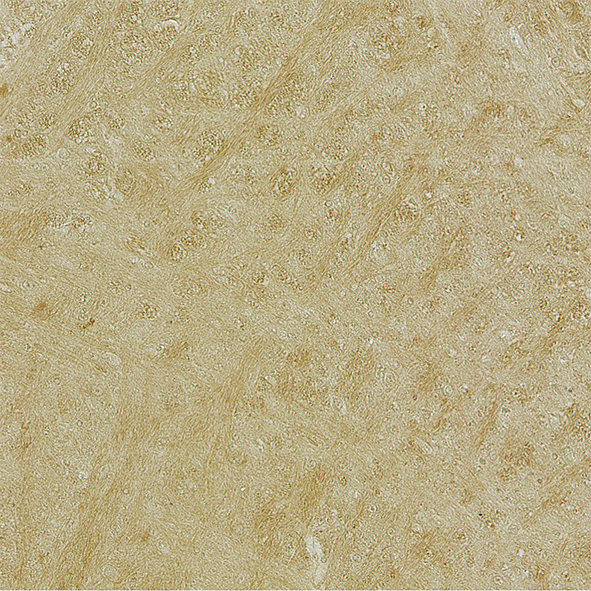

Supplement: Supplementary file 9 — Source data Fig. 6 [file 44321_2025_314_MOESM9_ESM.zip › Figure 6/D4_IDS.SWAP-ApoEco_Midbrain.tif]

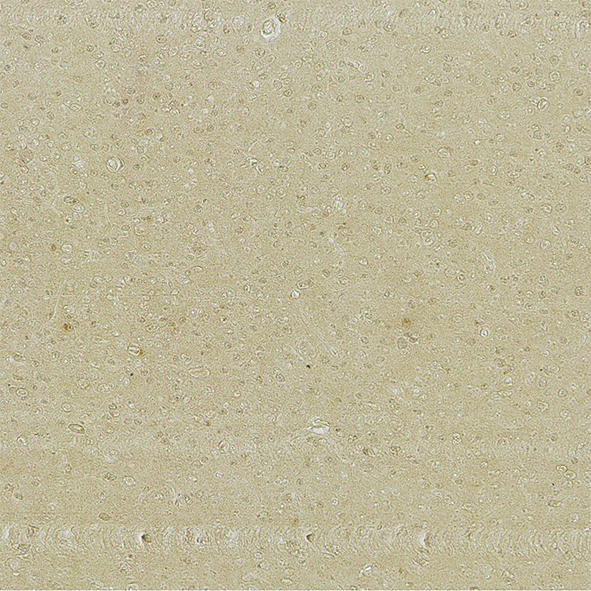

Supplement: Supplementary file 9 — Source data Fig. 6 [file 44321_2025_314_MOESM9_ESM.zip › Figure 6/A4_IDS.SWAP-ApoEco_Cortex.tif]

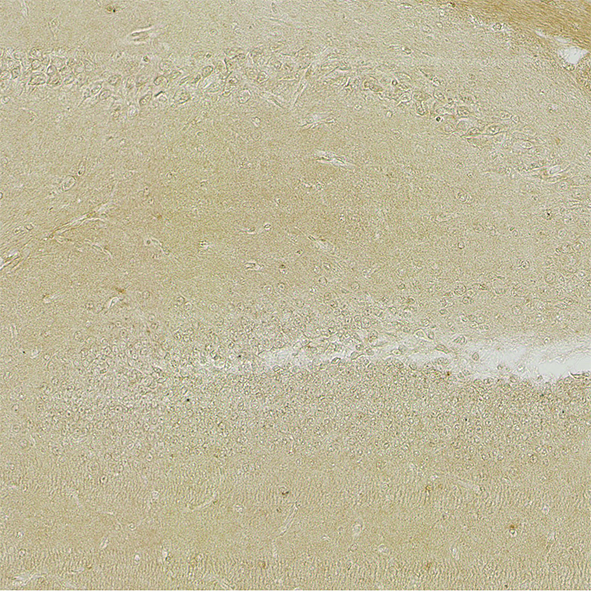

Supplement: Supplementary file 9 — Source data Fig. 6 [file 44321_2025_314_MOESM9_ESM.zip › Figure 6/B4_IDS.SWAP-ApoEco_Hippocampus.tif]

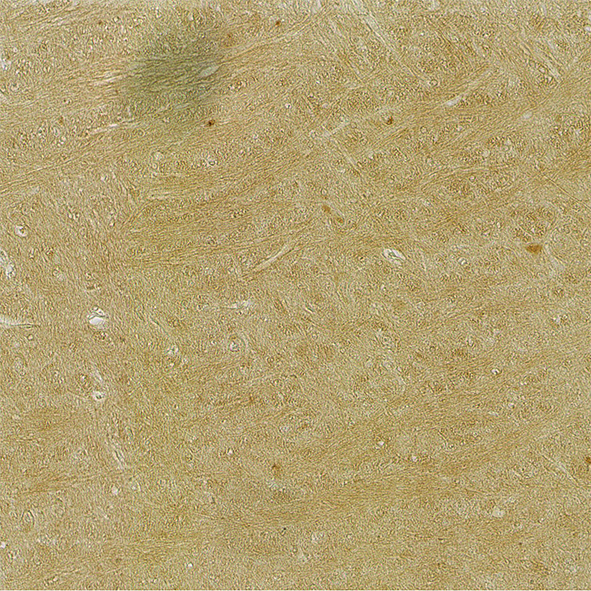

Supplement: Supplementary file 9 — Source data Fig. 6 [file 44321_2025_314_MOESM9_ESM.zip › Figure 6/F4_IDS.SWAP-ApoEco_Brainstem.tif]

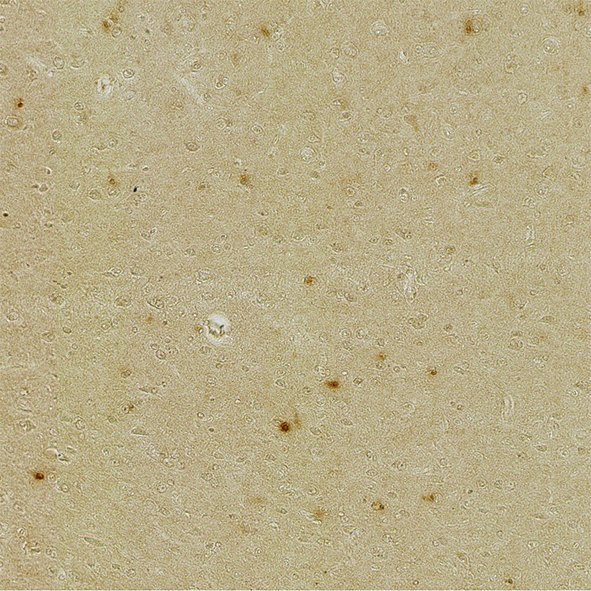

Supplement: Supplementary file 9 — Source data Fig. 6 [file 44321_2025_314_MOESM9_ESM.zip › Figure 6/A1_IDSco_Cortex.tif]
