# Supplementary figures and images for: Domain-substituted IGF2 tag modulates targeting of lentiviral gene therapy for Hunter syndrome (part 2 of 2)
Source: EMBO Mol Med. 2025 Sep 29;17(11):3197–226. doi: 10.1038/s44321-025-00314-3 (PMC12603107; doi:10.1038/s44321-025-00314-3)

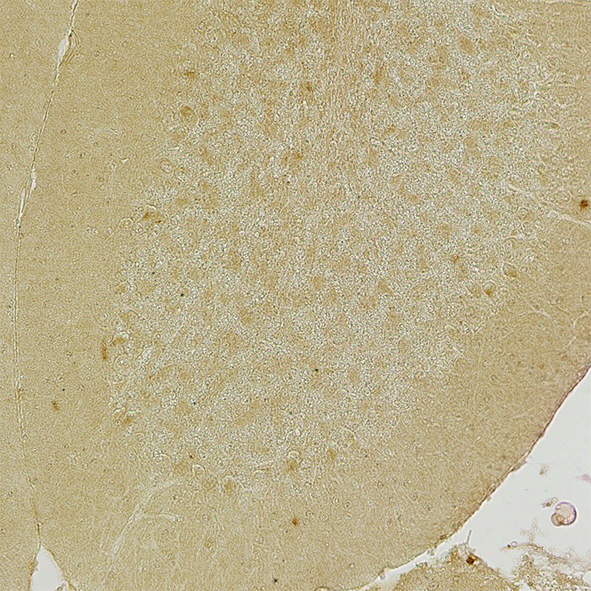

Supplement: Supplementary file 9 — Source data Fig. 6 [file 44321_2025_314_MOESM9_ESM.zip › Figure 6/E4_IDS.SWAP-ApoEco_Cerebellum.tif]

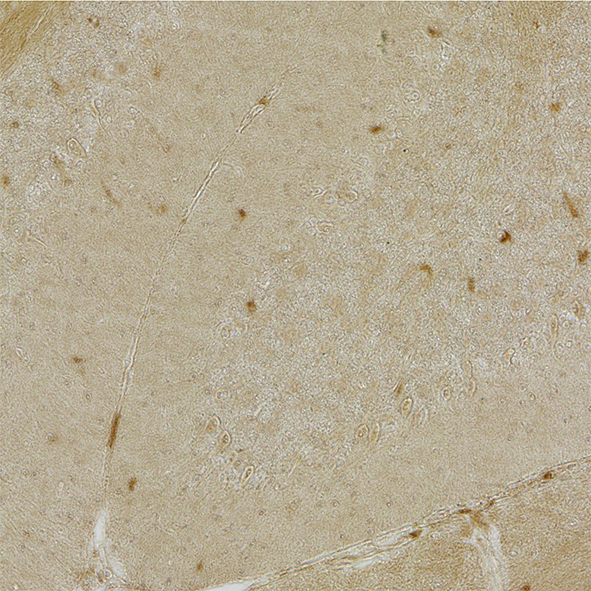

Supplement: Supplementary file 9 — Source data Fig. 6 [file 44321_2025_314_MOESM9_ESM.zip › Figure 6/E6_MPS II_Cerebellum.tif]

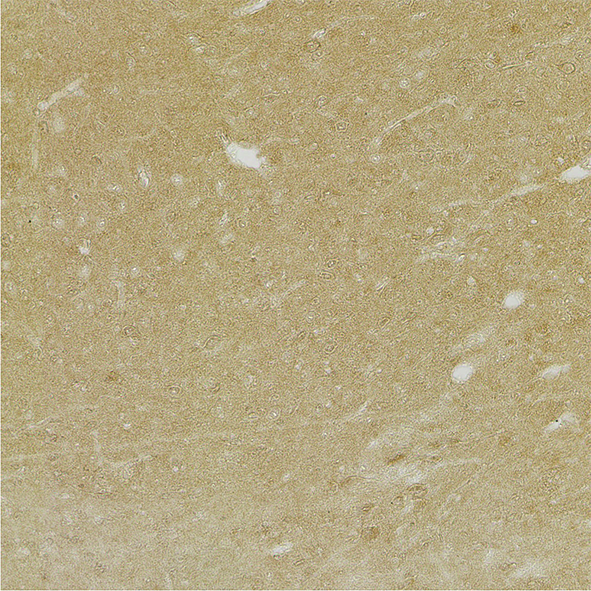

Supplement: Supplementary file 9 — Source data Fig. 6 [file 44321_2025_314_MOESM9_ESM.zip › Figure 6/F1_IDSco_Brainstem.tif]

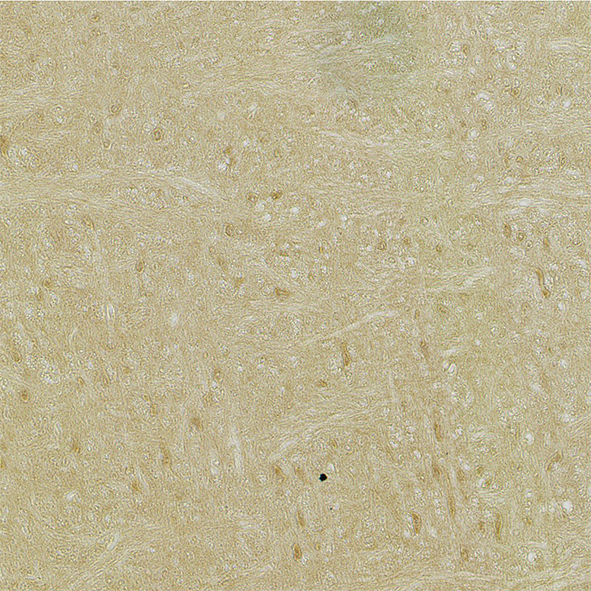

Supplement: Supplementary file 9 — Source data Fig. 6 [file 44321_2025_314_MOESM9_ESM.zip › Figure 6/F2_IDS.IGF2co_Brainstem.tif]

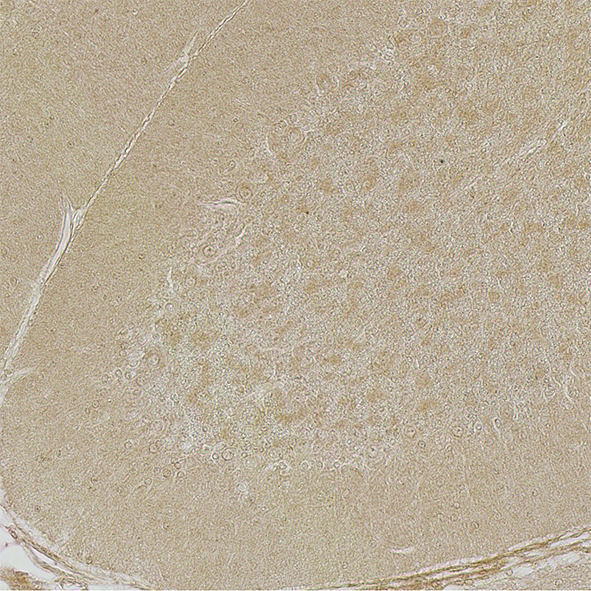

Supplement: Supplementary file 9 — Source data Fig. 6 [file 44321_2025_314_MOESM9_ESM.zip › Figure 6/E7_WT_Cerebellum.tif]

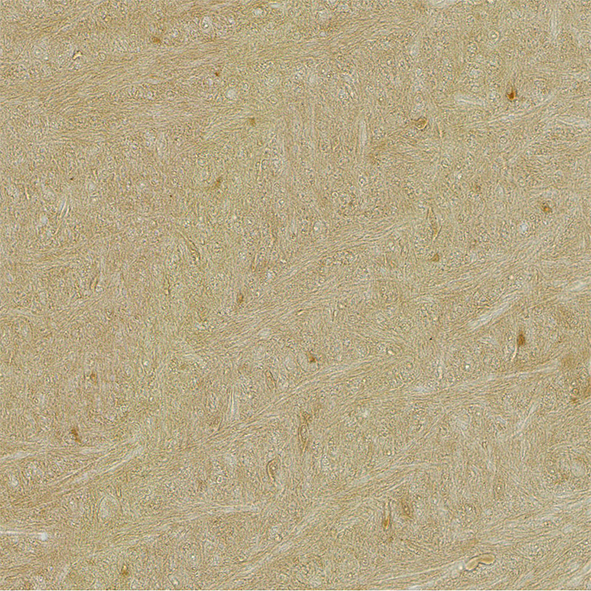

Supplement: Supplementary file 9 — Source data Fig. 6 [file 44321_2025_314_MOESM9_ESM.zip › Figure 6/F3_IDS.IGF2del_co_Brainstem.tif]

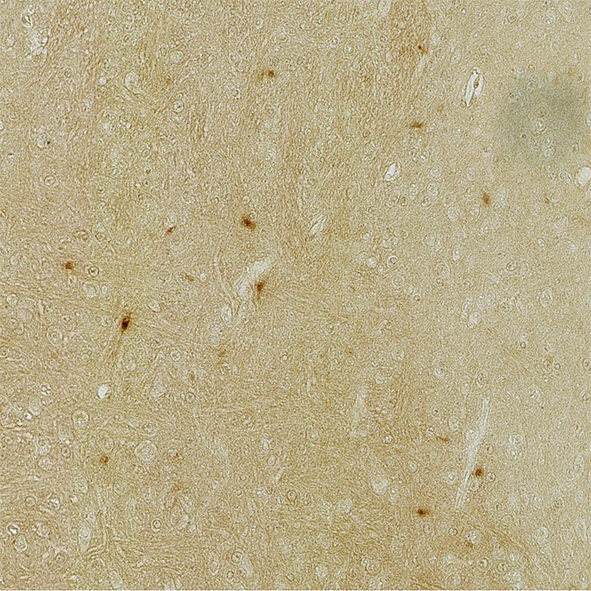

Supplement: Supplementary file 9 — Source data Fig. 6 [file 44321_2025_314_MOESM9_ESM.zip › Figure 6/A3_IDS.IGF2del_co_Cortex.tif]

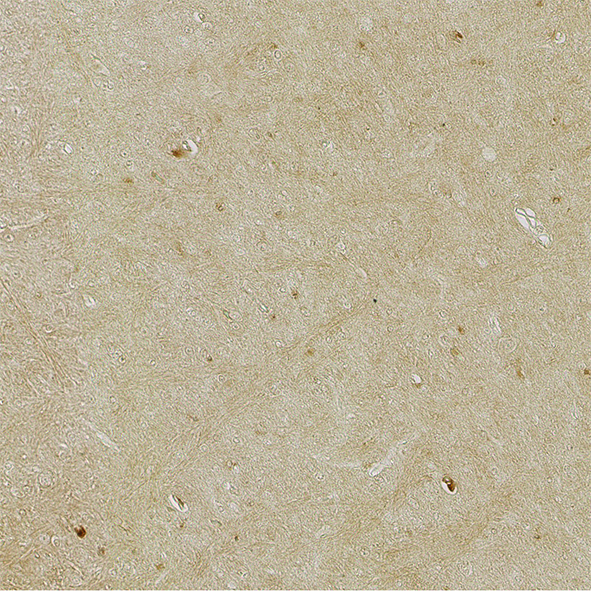

Supplement: Supplementary file 9 — Source data Fig. 6 [file 44321_2025_314_MOESM9_ESM.zip › Figure 6/D6_MPS II_Midbrain.tif]

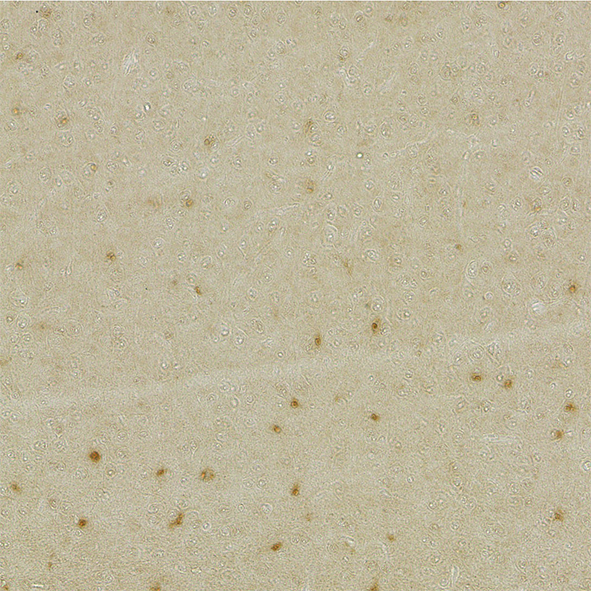

Supplement: Supplementary file 9 — Source data Fig. 6 [file 44321_2025_314_MOESM9_ESM.zip › Figure 6/A6_MPS II_Cortex.tif]

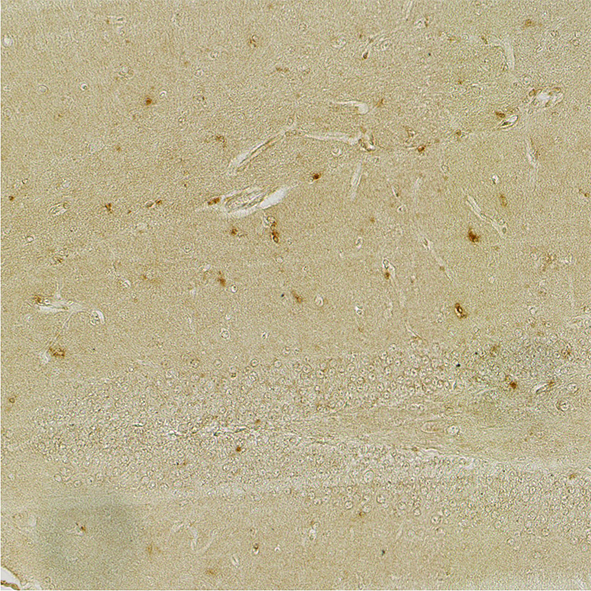

Supplement: Supplementary file 9 — Source data Fig. 6 [file 44321_2025_314_MOESM9_ESM.zip › Figure 6/B1_IDSco_Hippocampus.tif]

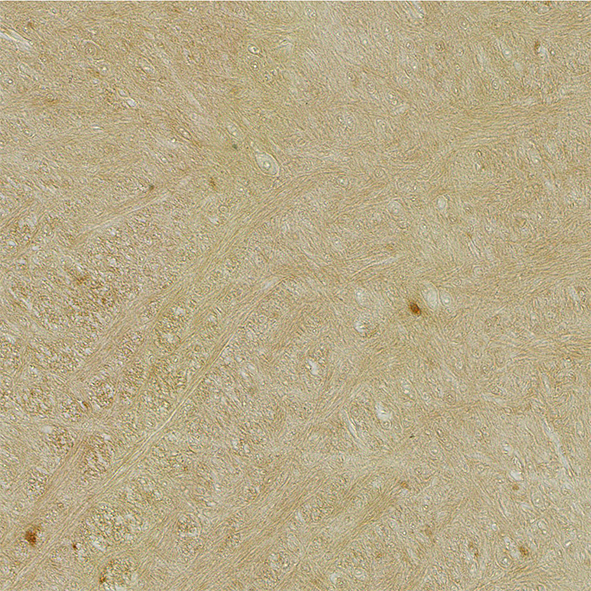

Supplement: Supplementary file 9 — Source data Fig. 6 [file 44321_2025_314_MOESM9_ESM.zip › Figure 6/D3_IDS.IGF2del_co_Midbrain.tif]

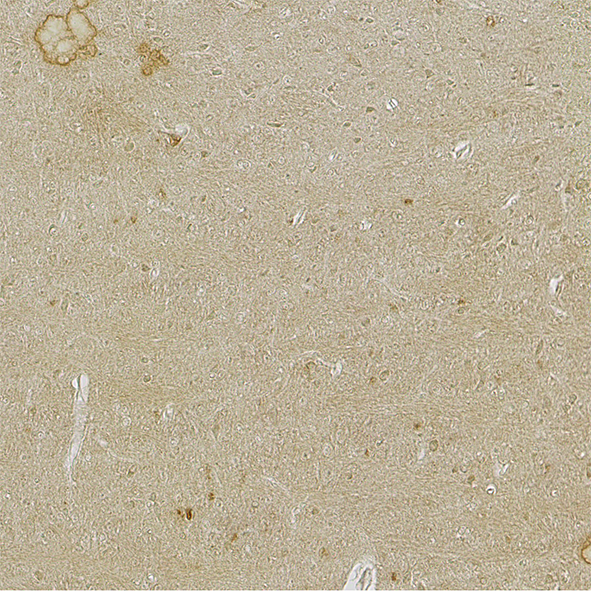

Supplement: Supplementary file 9 — Source data Fig. 6 [file 44321_2025_314_MOESM9_ESM.zip › Figure 6/F6_MPS II_Brainstem.tif]

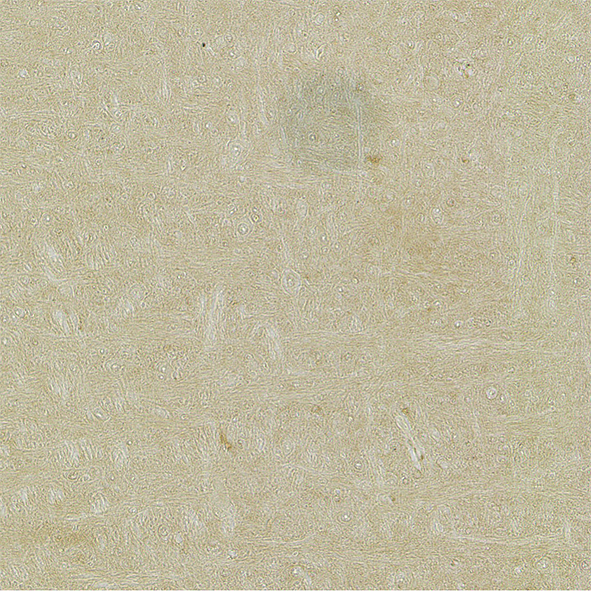

Supplement: Supplementary file 9 — Source data Fig. 6 [file 44321_2025_314_MOESM9_ESM.zip › Figure 6/D2_IDS.IGF2co_Midbrain.tif]

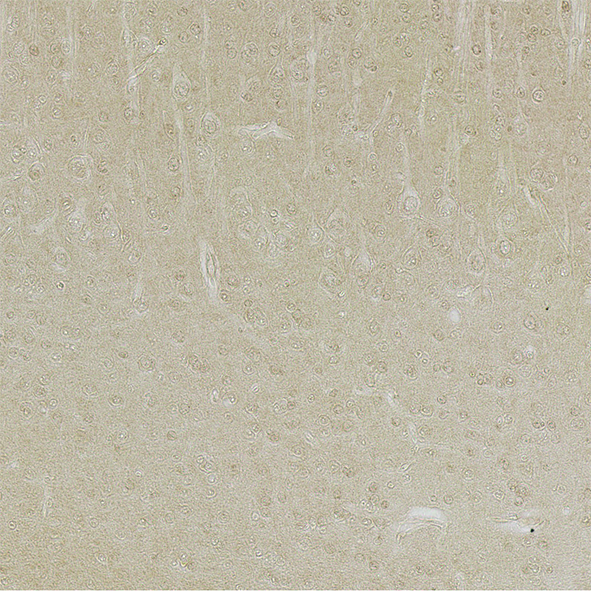

Supplement: Supplementary file 9 — Source data Fig. 6 [file 44321_2025_314_MOESM9_ESM.zip › Figure 6/A7_WT_Cortex.tif]

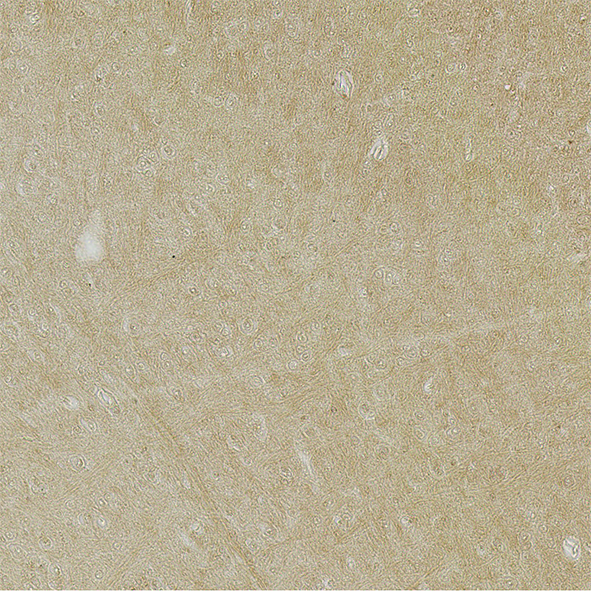

Supplement: Supplementary file 9 — Source data Fig. 6 [file 44321_2025_314_MOESM9_ESM.zip › Figure 6/D7_WT_Midbrain.tif]

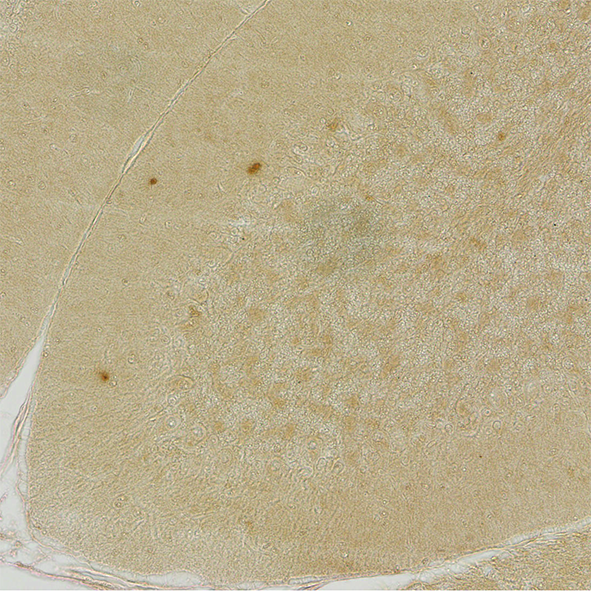

Supplement: Supplementary file 9 — Source data Fig. 6 [file 44321_2025_314_MOESM9_ESM.zip › Figure 6/E3_IDS.IGF2del_co_Cerebellum.tif]

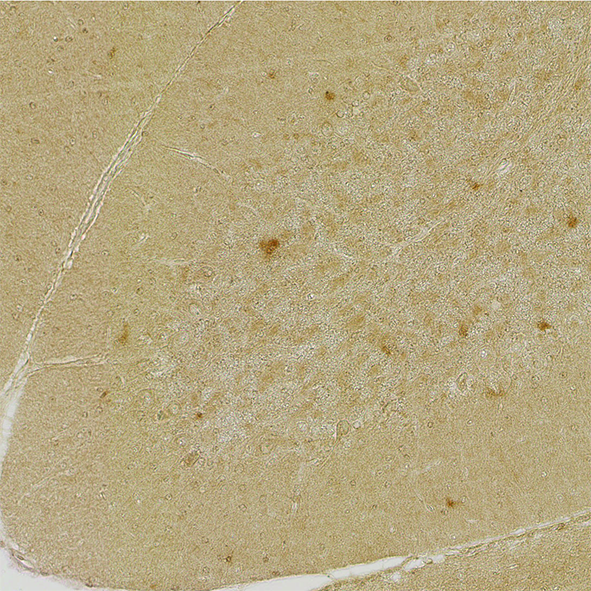

Supplement: Supplementary file 9 — Source data Fig. 6 [file 44321_2025_314_MOESM9_ESM.zip › Figure 6/E1_IDSco_Cerebellum.tif]

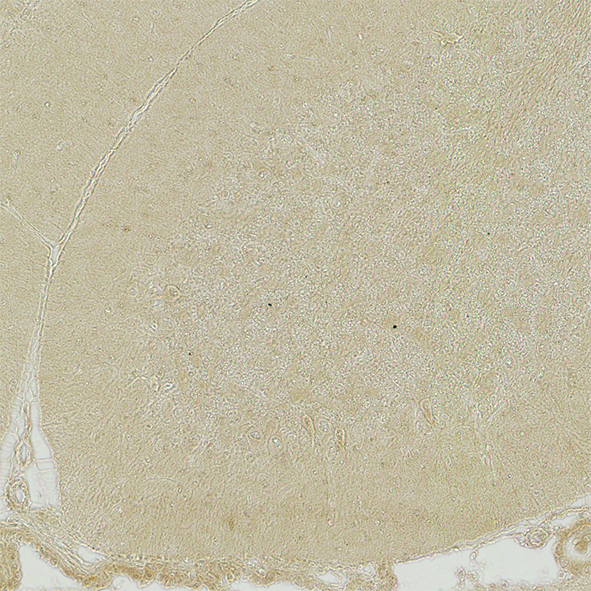

Supplement: Supplementary file 9 — Source data Fig. 6 [file 44321_2025_314_MOESM9_ESM.zip › Figure 6/E2_IDS.IGF2co_Cerebellum.tif]

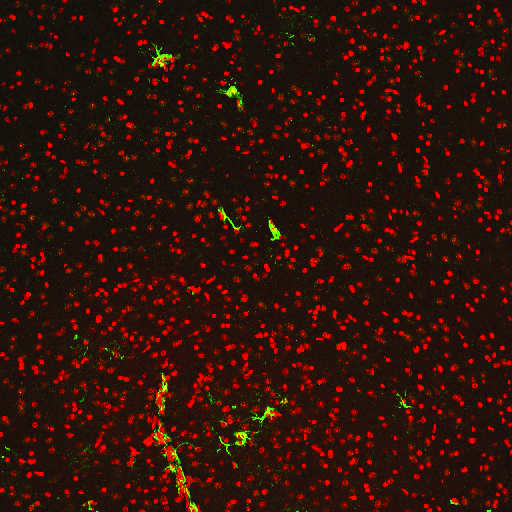

Supplement: Supplementary file 10 — Source data Fig. 7 [file 44321_2025_314_MOESM10_ESM.zip › Figure 7/C7_WT_Thalamus.tif]

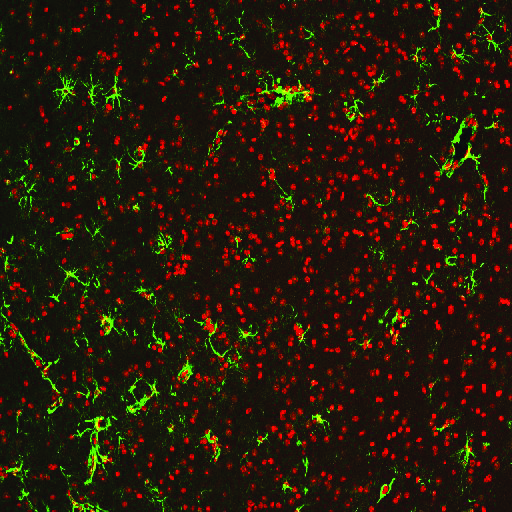

Supplement: Supplementary file 10 — Source data Fig. 7 [file 44321_2025_314_MOESM10_ESM.zip › Figure 7/C1_IDSco_Thalamus.tif]

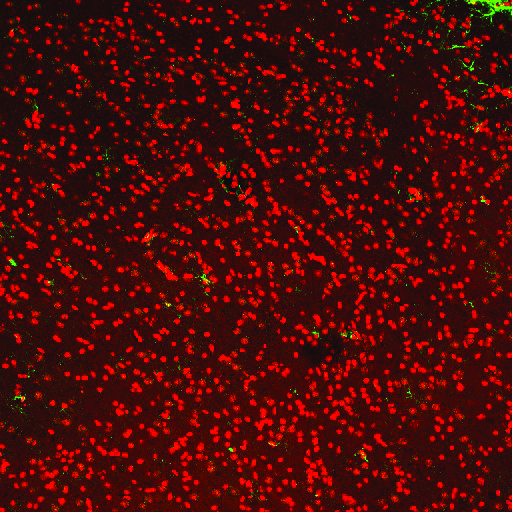

Supplement: Supplementary file 10 — Source data Fig. 7 [file 44321_2025_314_MOESM10_ESM.zip › Figure 7/C3_IDS.IGF2del_co_Thalamus.tif]

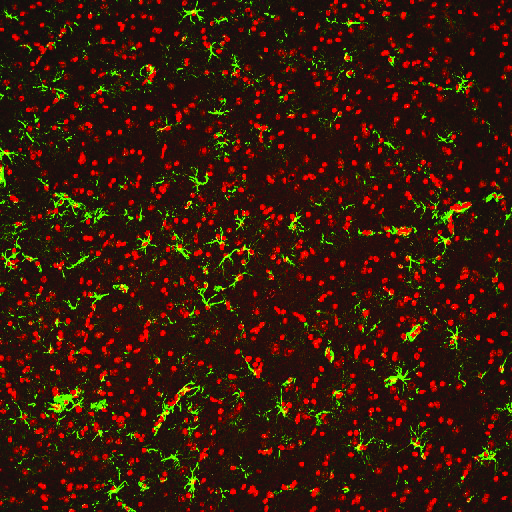

Supplement: Supplementary file 10 — Source data Fig. 7 [file 44321_2025_314_MOESM10_ESM.zip › Figure 7/C6_MPS II_Thalamus.tif]

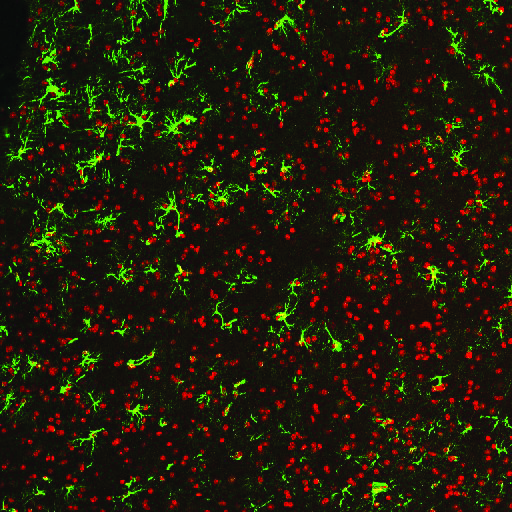

Supplement: Supplementary file 10 — Source data Fig. 7 [file 44321_2025_314_MOESM10_ESM.zip › Figure 7/D1_IDSco_Midbrain.tif]

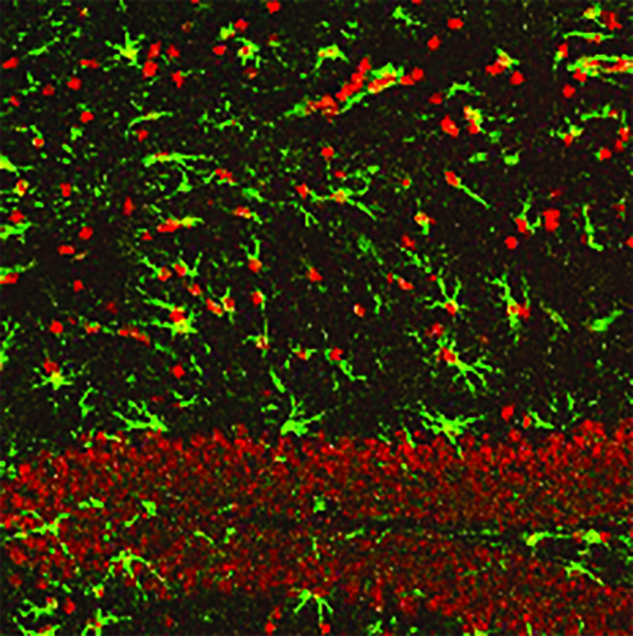

Supplement: Supplementary file 10 — Source data Fig. 7 [file 44321_2025_314_MOESM10_ESM.zip › Figure 7/B3_IDS.IGF2del_co_Hippocampus.tif]

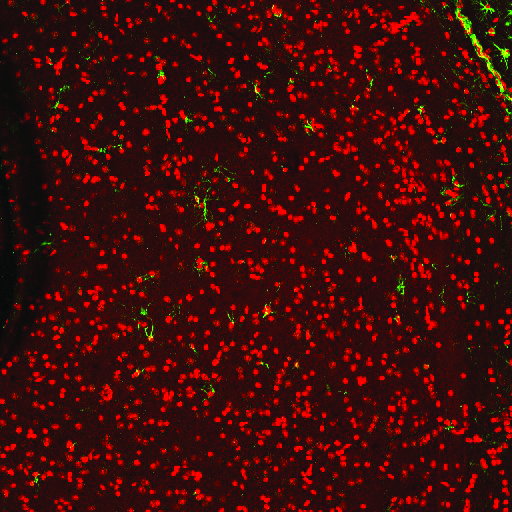

Supplement: Supplementary file 10 — Source data Fig. 7 [file 44321_2025_314_MOESM10_ESM.zip › Figure 7/C5_IDS.SWAP-RAP12x2_Thalamus.tif]

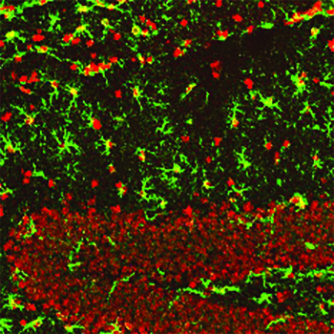

Supplement: Supplementary file 10 — Source data Fig. 7 [file 44321_2025_314_MOESM10_ESM.zip › Figure 7/B7_WT_Hippocampus.tif]

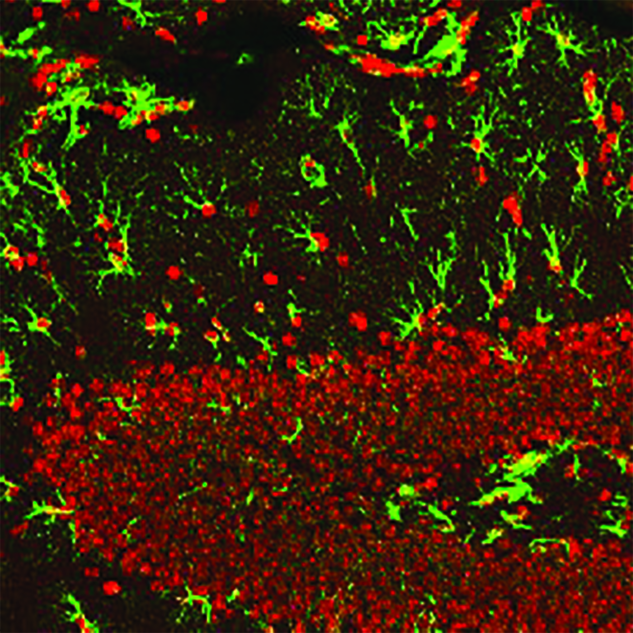

Supplement: Supplementary file 10 — Source data Fig. 7 [file 44321_2025_314_MOESM10_ESM.zip › Figure 7/B6_MPS II_Hippocampus.tif]

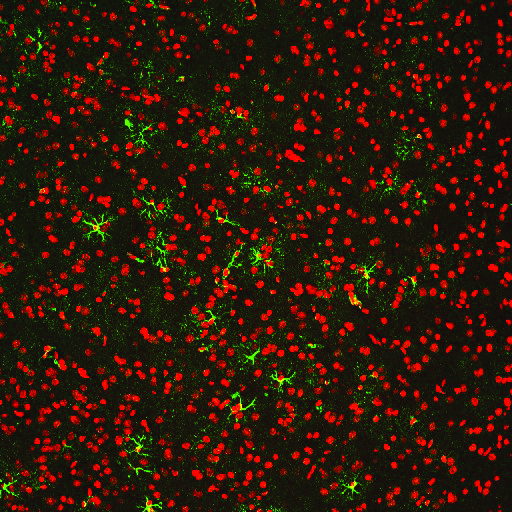

Supplement: Supplementary file 10 — Source data Fig. 7 [file 44321_2025_314_MOESM10_ESM.zip › Figure 7/A5_IDS.SWAP-RAP12x2_cortex.tif]

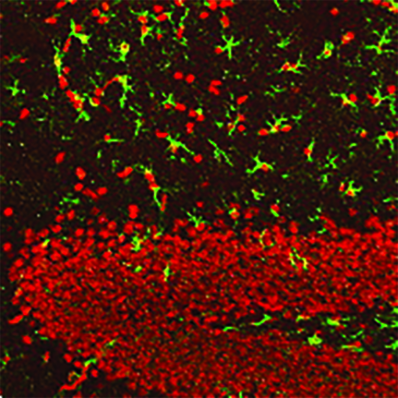

Supplement: Supplementary file 10 — Source data Fig. 7 [file 44321_2025_314_MOESM10_ESM.zip › Figure 7/B5_IDS.SWAP-RAP12x2_Hippocampus.tif]

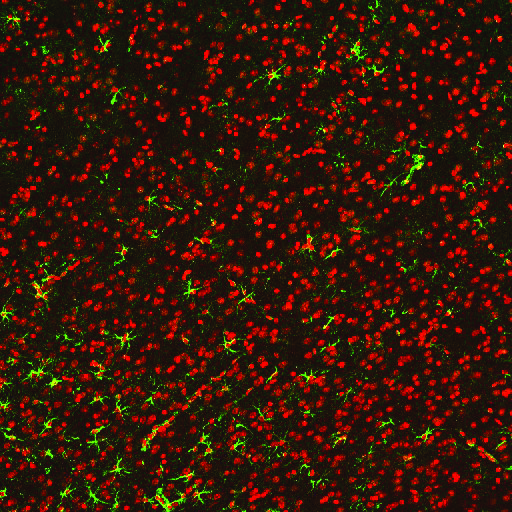

Supplement: Supplementary file 10 — Source data Fig. 7 [file 44321_2025_314_MOESM10_ESM.zip › Figure 7/A2_IDS.IGF2co_cortex.tif]

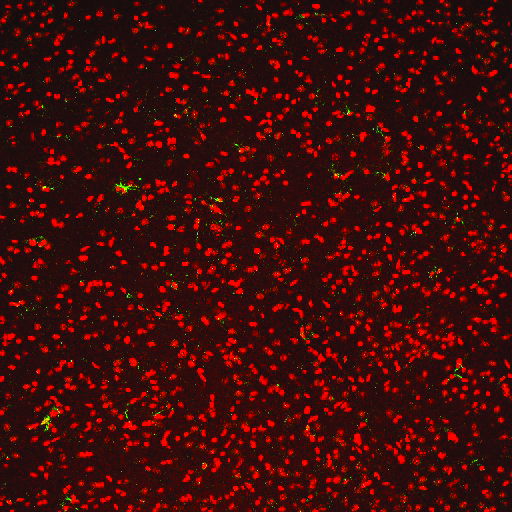

Supplement: Supplementary file 10 — Source data Fig. 7 [file 44321_2025_314_MOESM10_ESM.zip › Figure 7/C4_IDS.SWAP-ApoEco_Thalamus.tif]

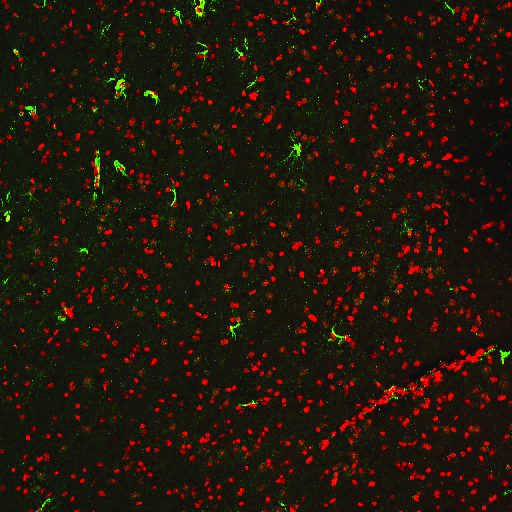

Supplement: Supplementary file 10 — Source data Fig. 7 [file 44321_2025_314_MOESM10_ESM.zip › Figure 7/C2_IDS.IGF2co_Thalamus.tif]

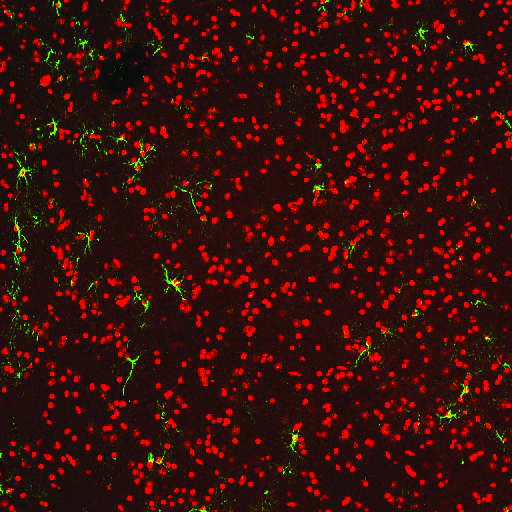

Supplement: Supplementary file 10 — Source data Fig. 7 [file 44321_2025_314_MOESM10_ESM.zip › Figure 7/D5_IDS.SWAP-RAP12x2_Midbrain.tif]

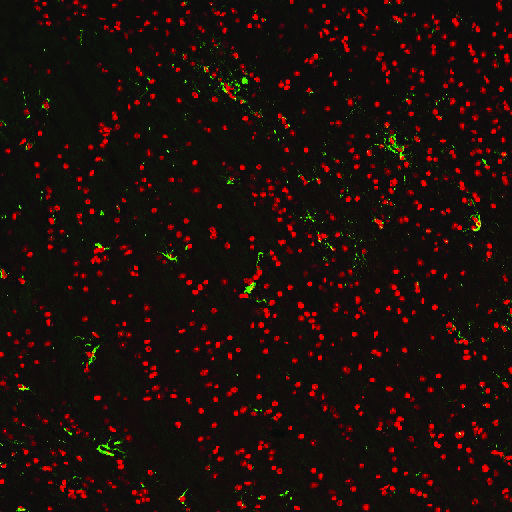

Supplement: Supplementary file 10 — Source data Fig. 7 [file 44321_2025_314_MOESM10_ESM.zip › Figure 7/D4_IDS.SWAP-ApoEco_Midbrain.tif]

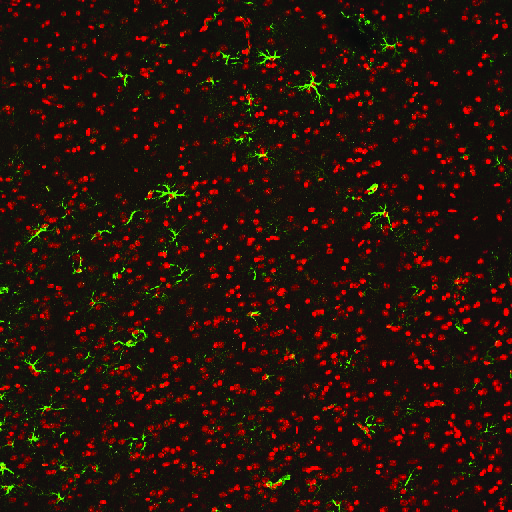

Supplement: Supplementary file 10 — Source data Fig. 7 [file 44321_2025_314_MOESM10_ESM.zip › Figure 7/A4_IDS.SWAP-ApoEco_cortex.tif]

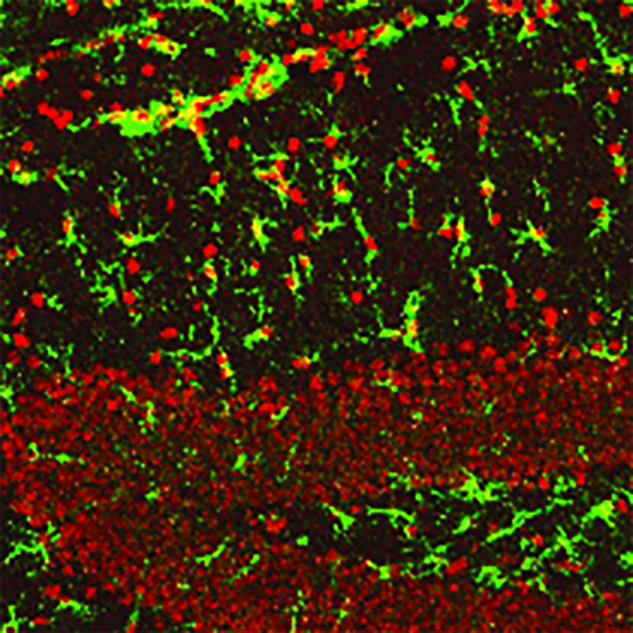

Supplement: Supplementary file 10 — Source data Fig. 7 [file 44321_2025_314_MOESM10_ESM.zip › Figure 7/B4_IDS.SWAP-ApoEco_Hippocampus.tif]

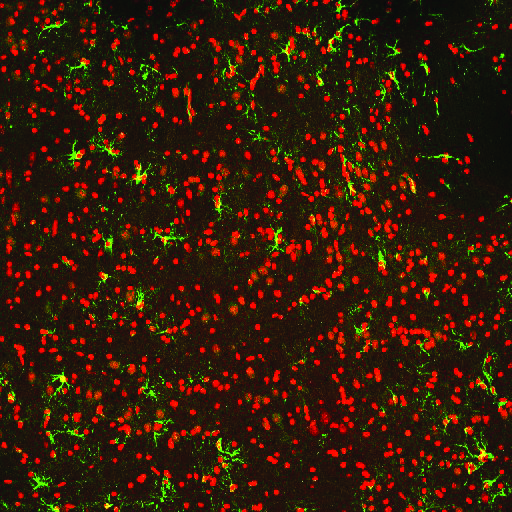

Supplement: Supplementary file 10 — Source data Fig. 7 [file 44321_2025_314_MOESM10_ESM.zip › Figure 7/E3_IDS.IGF2del_co_Brainstem.tif]

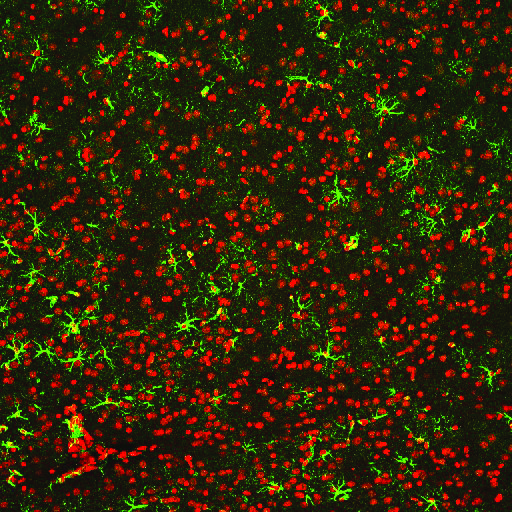

Supplement: Supplementary file 10 — Source data Fig. 7 [file 44321_2025_314_MOESM10_ESM.zip › Figure 7/A1_IDSco_cortex.tif]

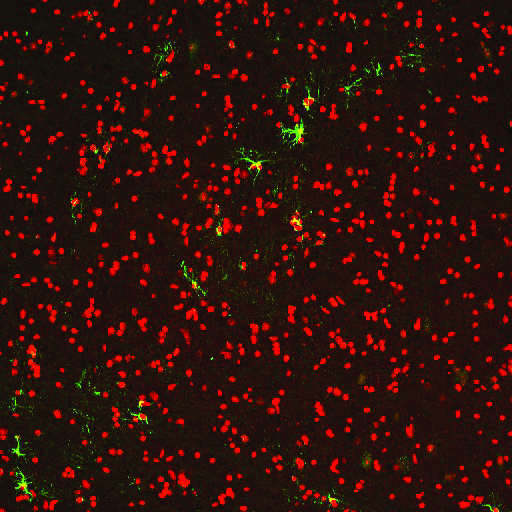

Supplement: Supplementary file 10 — Source data Fig. 7 [file 44321_2025_314_MOESM10_ESM.zip › Figure 7/E7_WT_Brainstem.tif]

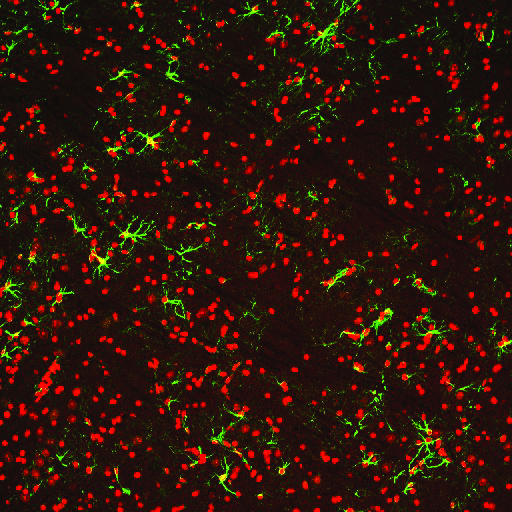

Supplement: Supplementary file 10 — Source data Fig. 7 [file 44321_2025_314_MOESM10_ESM.zip › Figure 7/E5_IDS.SWAP-RAP12x2_Brainstem.tif]

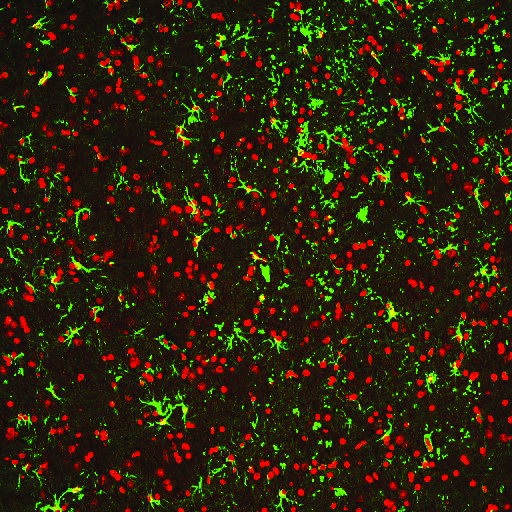

Supplement: Supplementary file 10 — Source data Fig. 7 [file 44321_2025_314_MOESM10_ESM.zip › Figure 7/E6_MPS II_Brainstem.tif]

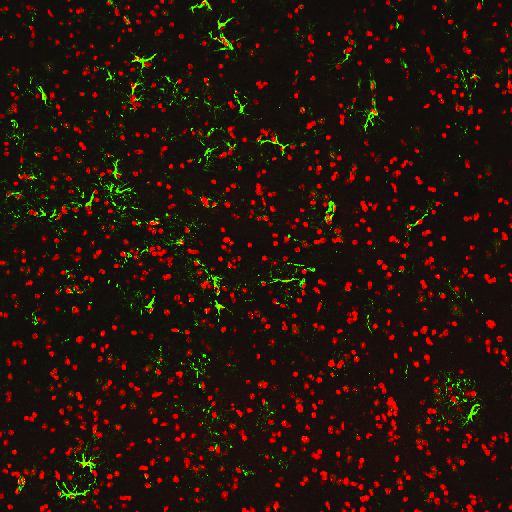

Supplement: Supplementary file 10 — Source data Fig. 7 [file 44321_2025_314_MOESM10_ESM.zip › Figure 7/E4_IDS.SWAP-ApoEco_Brainstem.tif]

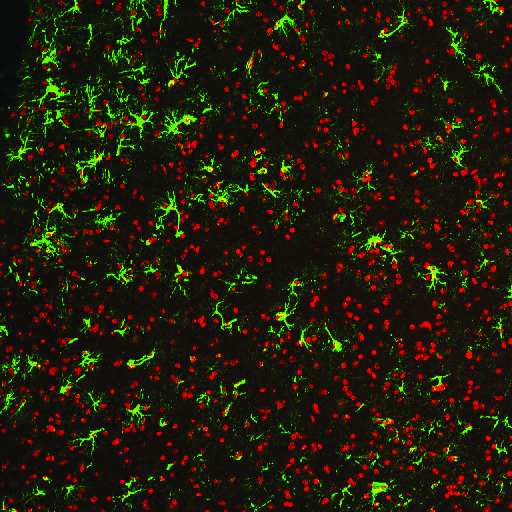

Supplement: Supplementary file 10 — Source data Fig. 7 [file 44321_2025_314_MOESM10_ESM.zip › Figure 7/E1_IDSco_Brainstem.tif]

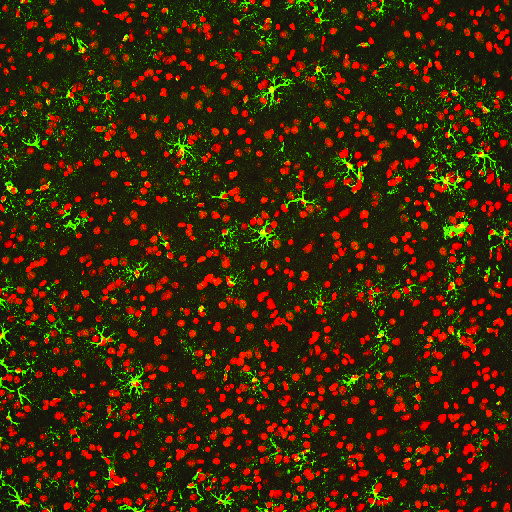

Supplement: Supplementary file 10 — Source data Fig. 7 [file 44321_2025_314_MOESM10_ESM.zip › Figure 7/A3_IDS.IGF2del_co_cortex.tif]

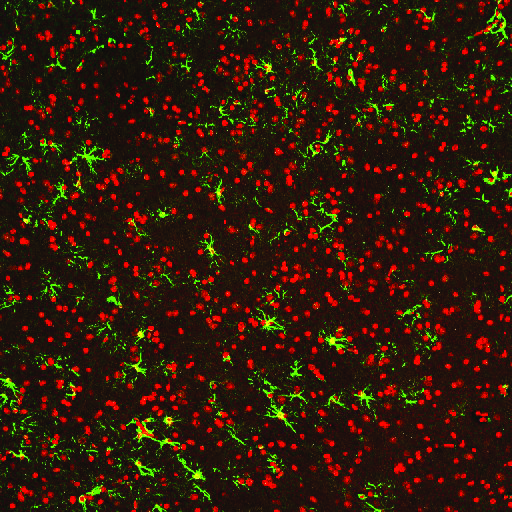

Supplement: Supplementary file 10 — Source data Fig. 7 [file 44321_2025_314_MOESM10_ESM.zip › Figure 7/D6_MPS II_Midbrain.tif]

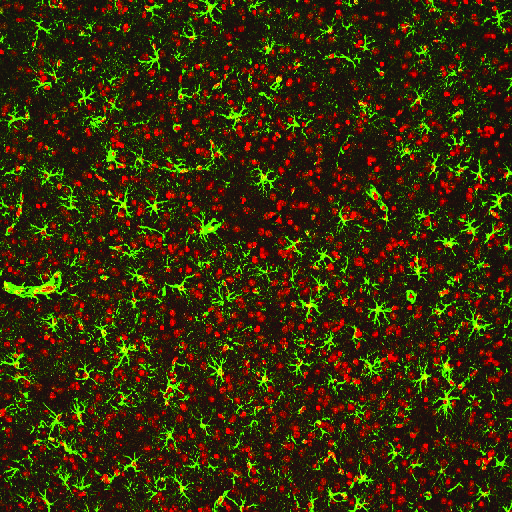

Supplement: Supplementary file 10 — Source data Fig. 7 [file 44321_2025_314_MOESM10_ESM.zip › Figure 7/A6_MPS II_cortex.tif]

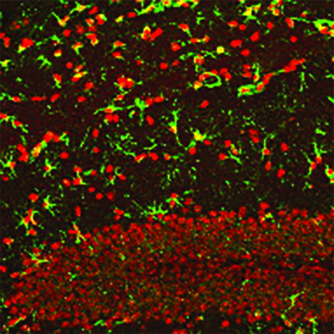

Supplement: Supplementary file 10 — Source data Fig. 7 [file 44321_2025_314_MOESM10_ESM.zip › Figure 7/B1_IDSco_Hippocampus.tif]

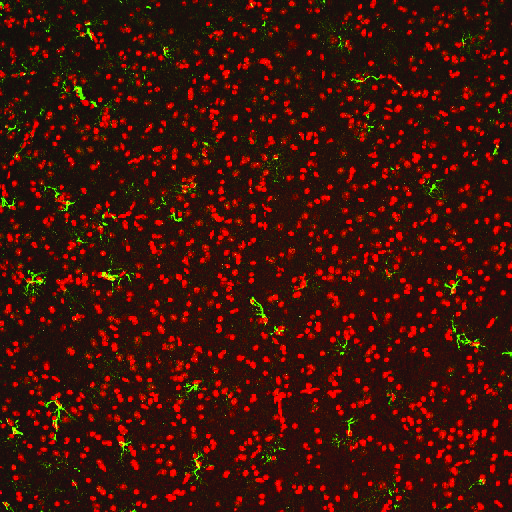

Supplement: Supplementary file 10 — Source data Fig. 7 [file 44321_2025_314_MOESM10_ESM.zip › Figure 7/D3_IDS.IGF2del_co_Midbrain.tif]

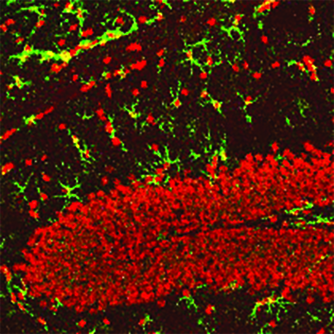

Supplement: Supplementary file 10 — Source data Fig. 7 [file 44321_2025_314_MOESM10_ESM.zip › Figure 7/B2_IDS.IGF2co_Hippocampustif.tif]

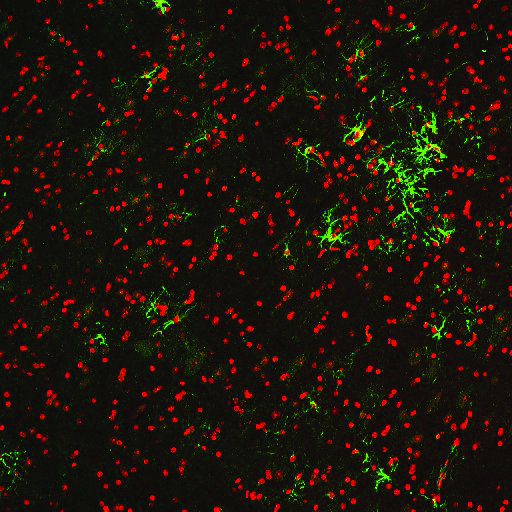

Supplement: Supplementary file 10 — Source data Fig. 7 [file 44321_2025_314_MOESM10_ESM.zip › Figure 7/E2_IDS.IGF2co_Brainstem.tif]

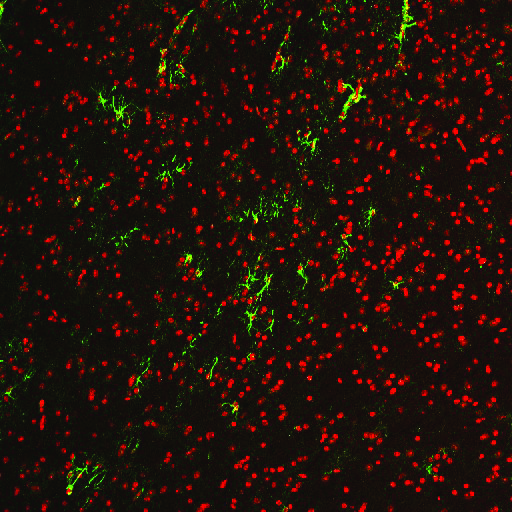

Supplement: Supplementary file 10 — Source data Fig. 7 [file 44321_2025_314_MOESM10_ESM.zip › Figure 7/D2_IDS.IGF2co_Midbrain.tif]

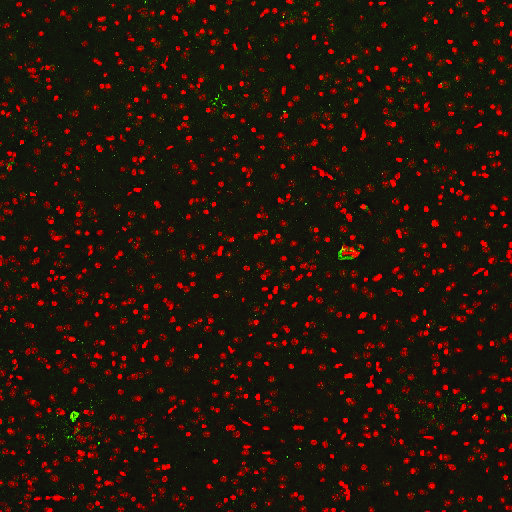

Supplement: Supplementary file 10 — Source data Fig. 7 [file 44321_2025_314_MOESM10_ESM.zip › Figure 7/A7_WT_cortex.tif]

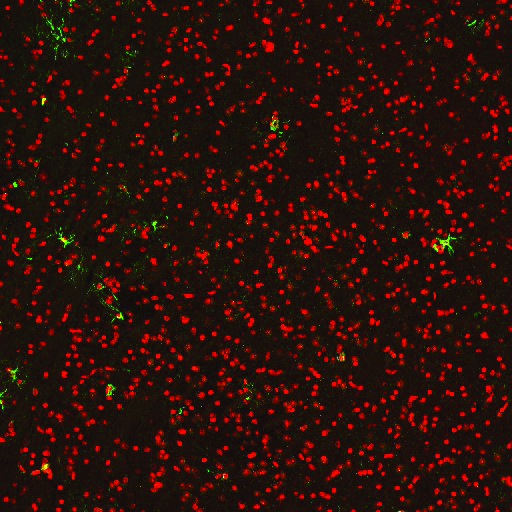

Supplement: Supplementary file 10 — Source data Fig. 7 [file 44321_2025_314_MOESM10_ESM.zip › Figure 7/D7_WT_Midbrain.tif]
