# Supplementary material for: Domain-substituted IGF2 tag modulates targeting of lentiviral gene therapy for Hunter syndrome
Source: EMBO Mol Med. 2025 Sep 29;17(11):3197–226. doi: 10.1038/s44321-025-00314-3 (PMC12603107; doi:10.1038/s44321-025-00314-3)
Supplement: Supplementary file 11 — Expanded View Figures [file 44321_2025_314_MOESM11_ESM.pdf]

Expanded View Figures

Figure EV1. IDS.SWAP processing and secretion.

(**A**, **B**) Binding of IDS.IGF2del to CI-M6P/IGF2R and IR-A was assessed using two methods: a direct CI-M6P/IGF2R ELISA (**A**) and a competitive IR-A ELISA using biotinylated insulin (**B**). (**C**) Cellular uptake test of SWAP variants containing an ApoE insert into MPS II fibroblasts (24-h uptake). IDS.IGF2 and IDS.ApoEII refer to versions of the IDS protein tagged at the C-terminus with either IGF2 or ApoEII (tandem repeat of ApoE) tags via a flexible linker, as previously described (Gleitz et al, 2018; Catalano et al, 2023). IDS.SWAP-ApoE_No Cys refers to a SWAP version without a cysteine residue at each end of the insertion. IDS.SWAP-ApoE_with Cys refers to a SWAP version with a cysteine residue at each end of the insertion. The adjusted P values were as follows: IDS.IGF2 vs. IDS.ApoEII $P = <0.0001$, IDS.ApoEII vs. IDS.SWAP-ApoE_No Cys $P = 0.0085$, IDS.ApoEII vs. IDS.SWAP-ApoE_with Cys $P = 0.0004$, IDS.SWAP-ApoE_No Cys vs. IDS.SWAP-ApoE_with Cys $P = 0.0395$. (**D**) Immunoblot analysis of IDS proteins using an anti-IDS antibody. The upper panel shows SDS-PAGE analysis of the cell lysate of HMC3 cells 10 days after transduction at MOI 13.5 with lentiviral vectors encoding the indicated fusion proteins. The lower panel shows SDS-PAGE analysis of the medium supernatant of HMC3 transduced as above. Both upper and lower panels were separated to eliminate an irrelevant condition. Medium was refreshed 24 h before sample collection. HMC3 cells transduced with a GFP vector and 0.1 µg of Elaprase served as negative and positive controls, respectively, during SDS-PAGE analysis. (**E**) Protein load for the western blot in Fig. EV1D and relative quantification (shown in Fig. 1C). Bottom panel was separated to eliminate an irrelevant condition. The stain free signal was quantified from the same gel used for immunoblot analysis to determine the total protein load. (**F**) Immunoblot analysis of IDS protein in five 2-fold dilutions of medium supernatant from HMC3 transduced with the indicated lentiviral vectors. 1 µg of Elaprase served as control. This was used to measure specific IDS activity in Fig. 1E. Lower panel was separated to eliminate an irrelevant condition. (**G**) Secreted IDS protein activity per VCN after transduction of HMC3 cells at MOI 5 and MOI 50. The adjusted p value for *MOI 5* IDS.IGF2 vs. IDS.SWAP-ApoE was 0.0283. Data information: data are presented as means ± SD. In (**C**), a total of 8 conditions were analyzed by one-way ANOVA; significant pairwise differences among the 4 relevant conditions are reported. In (**G**) data were analyzed by two-way ANOVA followed by Bonferroni's multiple testing correction. In (**A–C**) $n = 2$. In (**G**) $n = 3$. $^*P \leq 0.05$; $^{**}P \leq 0.01$; $^{***}P \leq 0.001$; $^{****}P \leq 0.0001$. Significant comparisons are indicated by brackets.

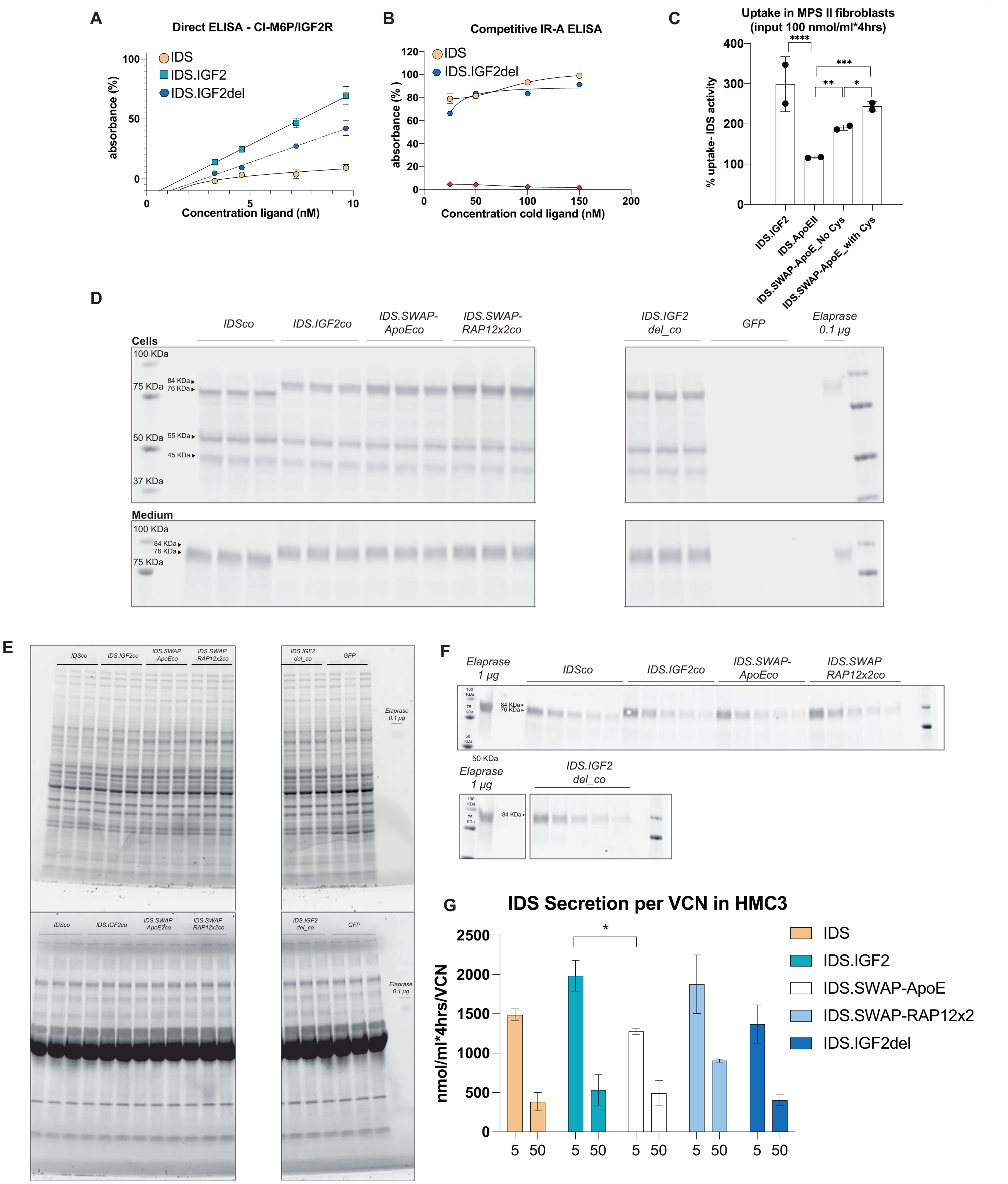
A
Direct ELISA - CI-M6P/IGF2R
IDS
IDS.IGF2
IDS.IGF2del
absorbance (%)
Concentration ligand (nM)
B
Competitive IR-A ELISA
IDS
IDS.IGF2del
absorbance (%)
Concentration cold ligand (nM)
C
Uptake in MPS II fibroblasts
(input 100 nmol/ml*4hrs)
% uptake- IDS activity
IDS.IGF2
IDS.ApoEII
IDS.SWAP-ApoE_No Cys
IDS.SWAP-ApoE_with Cys
D
IDSco
IDS.IGF2co
IDS.SWAP-ApoEco
IDS.SWAP-RAP12x2co
IDS.IGF2 del_co
GFP
Elaprase 0.1 µg
Cells
Medium
E
F
Elaprase 1 µg
IDSco
IDS.IGF2co
IDS.SWAP-ApoEco
IDS.SWAP RAP12x2co
IDS.IGF2 del_co
G
IDS Secretion per VCN in HMC3
nmol/ml*4hrs/VCN
IDS
IDS.IGF2
IDS.SWAP-ApoE
IDS.SWAP-RAP12x2
IDS.IGF2del

 © The Author(s)

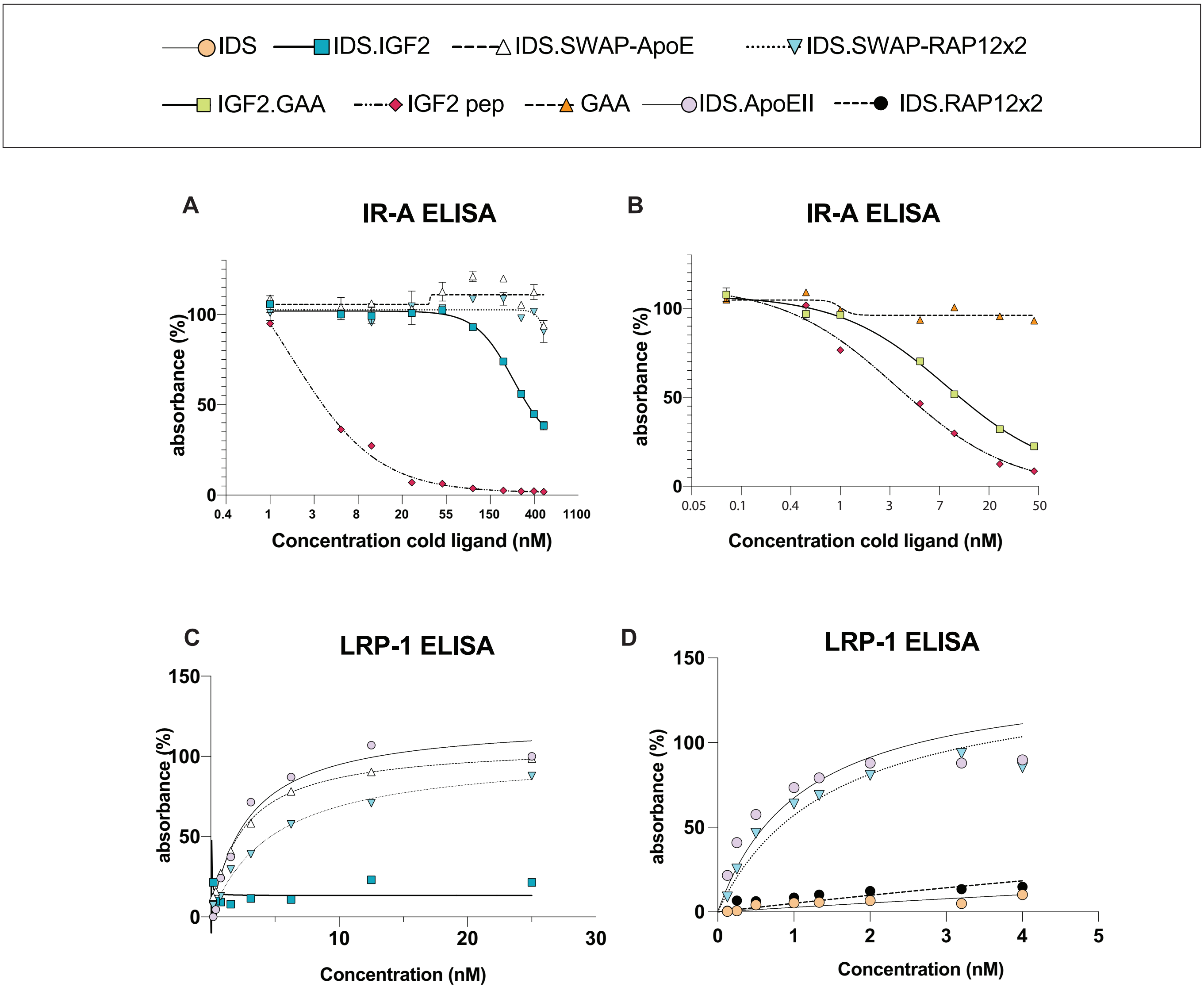

Figure EV2. CI-M6P/IGF2IGF2R, IR-A, LRP-1 ELISAs.

(**A**, **B**) competitive IR-A ELISA using biotinylated insulin. Concentrations of competing ligands are indicated. Ligands were derived from conditioned media produced in HEK 293T cells. (**C**, **D**) direct LRP-1 ELISA. Concentrations of ligands are indicated. Data information: data are means ± SD. Regression analysis is shown in Table EV1. $n = 2$ (**A–C**) or $n = 1$ (**D**).

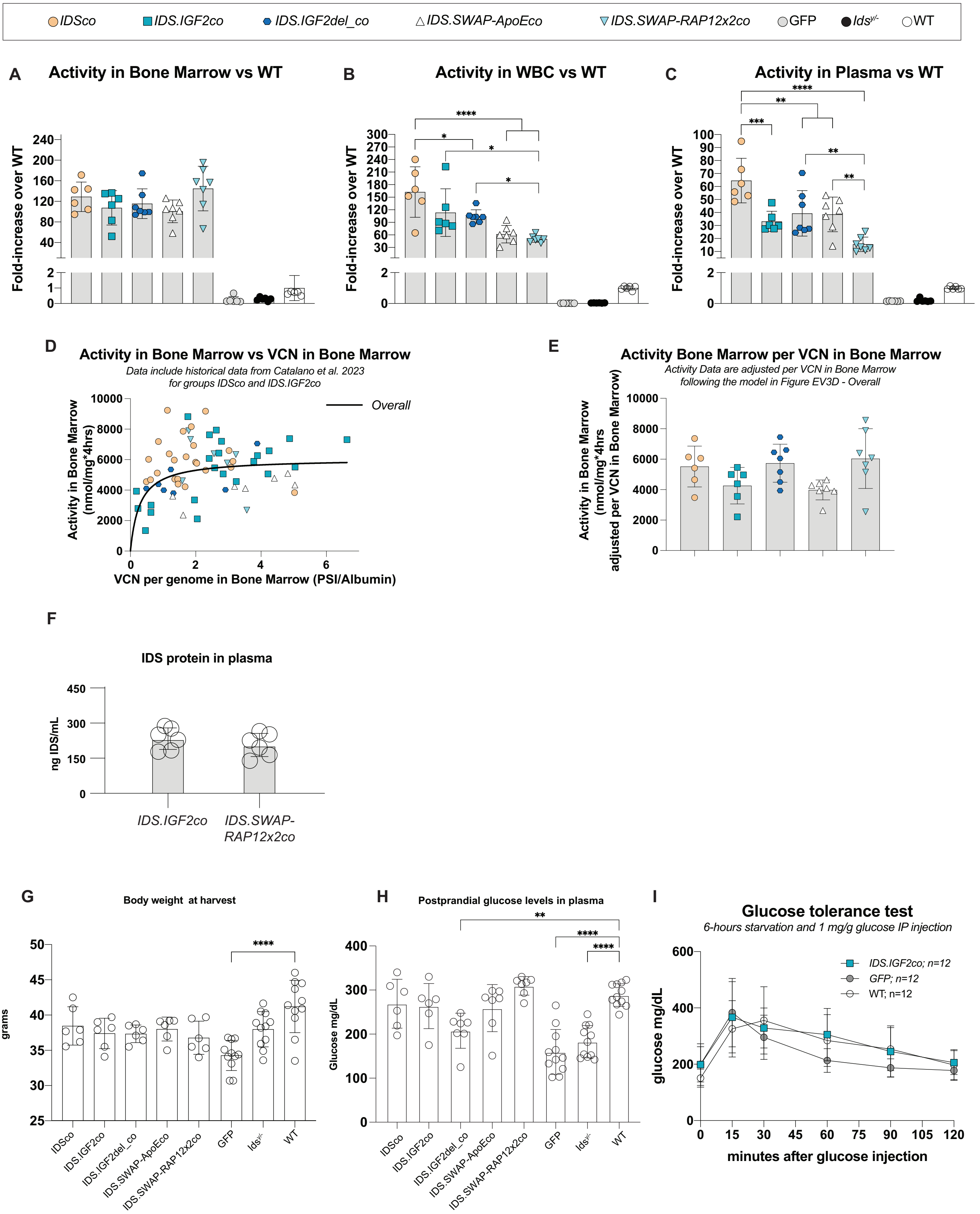
IDSco
IDS.IGF2co
IDS.IGF2del_co
IDS.SWAP-ApoEco
IDS.SWAP-RAP12x2co
GFP
Ids^y/-
WT
A
Activity in Bone Marrow vs WT
Fold-increase over WT
B
Activity in WBC vs WT
Fold-increase over WT
C
Activity in Plasma vs WT
Fold-increase over WT
D
Activity in Bone Marrow vs VCN in Bone Marrow
Data include historical data from Catalano et al. 2023 for groups IDSco and IDS.IGF2co
Overall
Activity in Bone Marrow (nmol/mg*4hrs)
VCN per genome in Bone Marrow (PSI/Albumin)
E
Activity Bone Marrow per VCN in Bone Marrow
Activity Data are adjusted per VCN in Bone Marrow following the model in Figure EV3D - Overall
Activity in Bone Marrow (nmol/mg*4hrs adjusted per VCN in Bone Marrow)
F
IDS protein in plasma
ng IDS/mL
IDS.IGF2co
IDS.SWAP-RAP12x2co
G
Body weight at harvest
grams
H
Postprandial glucose levels in plasma
Glucose mg/dL
I
Glucose tolerance test
6-hours starvation and 1 mg/g glucose IP injection
IDS.IGF2co; n=12
GFP; n=12
WT; n=12
glucose mg/dL
minutes after glucose injection

 © The Author(s)

Figure EV3. Supraphysiological levels of IDS activity in hematological samples, body weight, and glucose homeostasis.

(**A–C**) IDS enzyme activity in hematological tissues. Fold-increase versus WT in bone marrow (**A**), WBC (**B**), and plasma (**C**). In (**B**, **C**) the adjusted *P* values were as follows: (**B**) *IDSco* vs. *IDS.SWAP-ApoEco* $P = <0.0001$, *IDSco* vs. *IDS.SWAP-RAP12x2co* $P = <0.0001$, *IDSco* vs. *IDS.IGF2del_co* $P = 0.0317$, *IDS.IGF2co* vs. *IDS.SWAP-RAP12x2* $P = 0.0146$, *IDS.IGF2del_co* vs. *IDS.SWAP-RAP12x2* $P = 0.0492$. (**C**) *IDSco* vs. *IDS.IGF2co* $P = 0.0002$, *IDSco* vs. *IDS.IGF2del_co* $P = 0.0030$, *IDSco* vs. *IDS.SWAP-ApoEco* $P = 0.0020$, *IDSco* vs. *IDS.SWAP-RAP12x2co* $P = <0.0001$, *IDS.IGF2del_co* vs. *IDS.SWAP-RAP12x2co* $P = 0.0038$, *IDS.SWAP-ApoEco* vs. *IDS.SWAP-RAP12x2co* $P = 0.0058$. (**D**, **E**) Relationship between IDS activity in bone marrow and VCN in bone marrow. (**F**) Sandwich ELISA to quantify IDS protein levels in plasma. (**G**) Body weight at harvest after gene therapy. The adjusted *P* value for *GFP* vs. WT was <0.0001. (**H**) Postprandial glucose levels at harvest. The adjusted *P* values were as follows: *IDS.IGF2del_co* vs. WT $P = 0.0015$, *GFP* vs. WT $P = <0.0001$, $Ids^{y/-}$ vs. WT $P = <0.0001$. (**I**) Glucose tolerance test. Data information: data are means ± SD. Regression analysis of (**D**) is shown in Table EV1. In (**A–C**, **E**, **G**, **H**) data were analyzed by one-way ANOVA followed by Bonferroni's multiple testing correction. In (**H**), comparisons were made against WT. In (**F**) data were analyzed by *t* test. In (**A–C**, **E**) $n = 6$ for *IDSco*, *IDS.IGF2co*, *GFP*, $Ids^{y/-}$, and *WT*, $n = 7$ for *IDS.IGF2del_co*, *IDS.SWAP-ApoEco*, *IDS.SWAP-RAP12x2co*. In (**F**) $n = 6$. In (**G**, **H**) for *IDSco*, *IDS.IGF2co*, *IDS.IGF2del_co*, *IDS.SWAP-ApoEco*, *IDS.SWAP-RAP12x2co* $n = 6$; for *GFP*, $Ids^{y/-}$, and *WT* $n = 11$. In (**I**) $n = 12$. *$P \le 0.05$; **$P \le 0.01$; ***$P \le 0.001$; ****$P \le 0.001$. Significant comparisons are indicated by brackets.

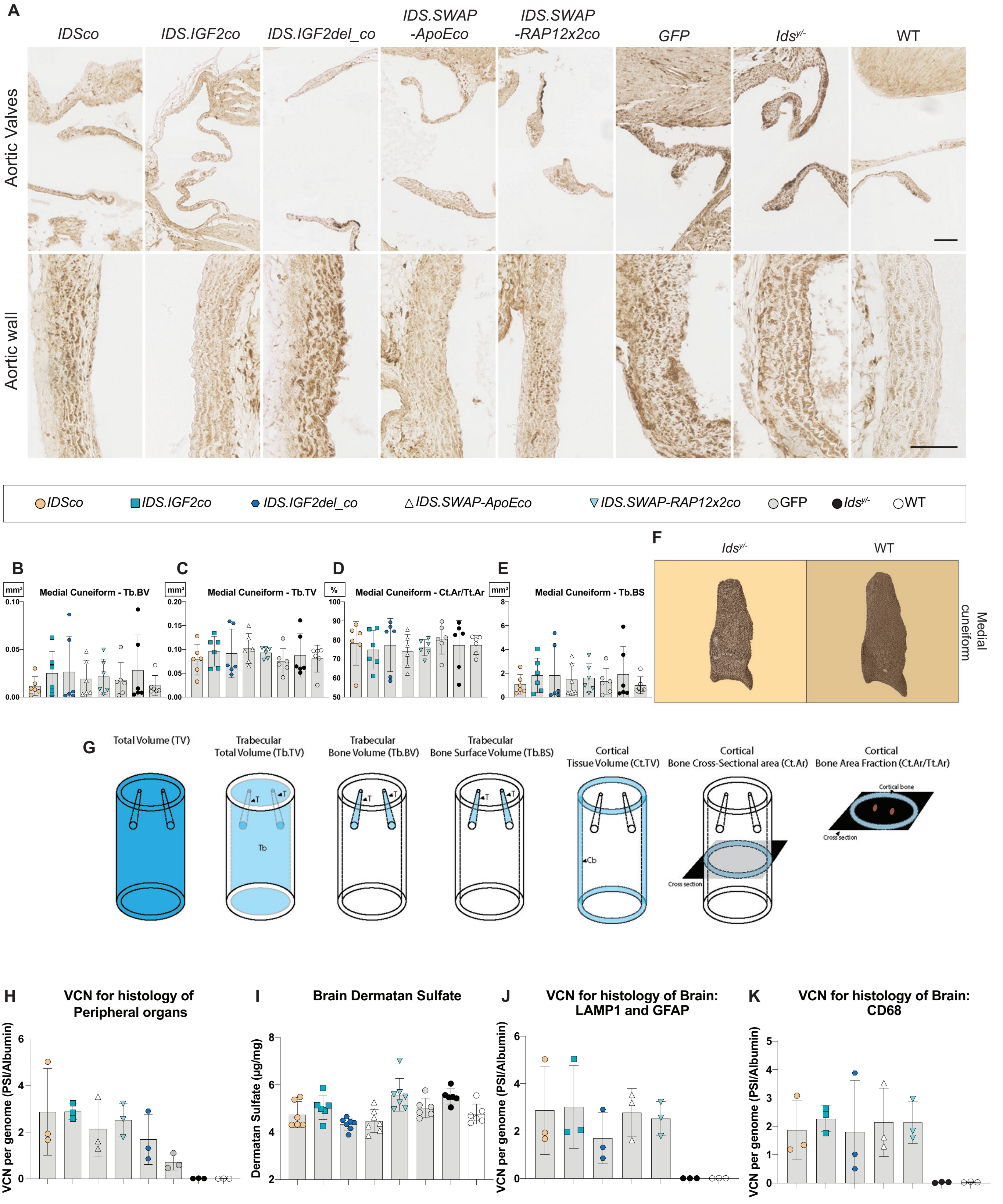
A
IDSco
IDS.IGF2co
IDS.IGF2del_co
IDS.SWAP -ApoEco
IDS.SWAP -RAP12x2co
GFP
Idsy/-
WT
Aortic Valves
Aortic wall
IDSco IDS.IGF2co IDS.IGF2del_co IDS.SWAP-ApoEco IDS.SWAP-RAP12x2co GFP Idsy/- WT
B Medial Cuneiform - Tb.BV
C Medial Cuneiform - Tb.TV
D Medial Cuneiform - Ct.Ar/Tt.Ar
E Medial Cuneiform - Tb.BS
F Idsy/- WT Medial cuneiform
G
Total Volume (TV)
Trabecular Total Volume (Tb.TV)
Trabecular Bone Volume (Tb.BV)
Trabecular Bone Surface Volume (Tb.BS)
Cortical Tissue Volume (Ct.TV)
Cortical Bone Cross-Sectional area (Ct.Ar)
Cortical Bone Area Fraction (Ct.Ar/Tt.Ar)
Cortical bone
Cross section
H VCN for histology of Peripheral organs
VCN per genome (PSI/Albumin)
I Brain Dermatan Sulfate
Dermatan Sulfate (µg/mg)
J VCN for histology of Brain: LAMP1 and GFAP
VCN per genome (PSI/Albumin)
K VCN for histology of Brain: CD68
VCN per genome (PSI/Albumin)

 © The Author(s)

Figure EV4. LAMP1 staining in heart, microarchitecture of the *medial cuneiform*, brain dermatan sulfate levels, and bone marrow VCN for histology.

(**A**) LAMP1 staining in heart valves and aortic wall. $n = 3$. Scale bar = 100 μm. (**B–E**) Microarchitecture analysis of the *medial cuneiform*. (**F**) 3D rendering of reconstructed μCT scans of the *medial cuneiform*. (**G**) Explanatory drawings of μCT parameters. The volume/area measured by the μCT parameters is highlighted in blue. (**H**) VCN per genome in bone marrow of mice used for histological analysis of peripheral tissues. (**I**) Dermatan sulfate levels in total brain homogenates measured by mass-spectrometry. (**J**, **K**) VCN per genome in bone marrow of mice used for immunostaining of LAMP1 and GFAP (**J**), or CD68 (**K**) in brain. Data information: data are presented as means ± SD and were analyzed by one-way ANOVA with Bonferroni's correction. In (**B–E**) $n = 6$; in (**H**, **J**, **K**) $n = 3$; in (**I**) $n = 6$ (*IDSco, IDS.IGF2co, GFP,* $Ids^{y/-}$, WT) or $n = 7$ (*IDS.IGF2del_co, IDS.SWAP-ApoEco, IDS.SWAP*-RAP12x2co). Tb: trabecular bone; T: trabeculae; Cb: cortical bone. Data are presented as means ± SD and were analyzed by one-way ANOVA with Bonferroni's correction.

© The Author(s)

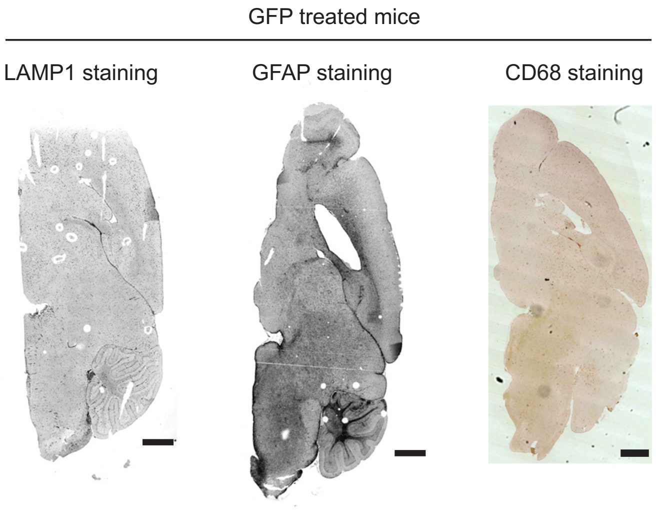

Figure EV5. Full scans of sagittal brain sections of GFP-treated mice stained for LAMP1, GFAP or CD68.

Example of sagittal brain sections from GFP-treated mice stained for LAMP1 (**left panel**), GFAP (**central panel**) or CD68 (**right panel**). $n = 3$. Scale bar = 1 mm.

 © The Author(s)
